# Antibody expressing pea seeds as fodder for prevention of gastrointestinal parasitic infections in chickens

**DOI:** 10.1186/1472-6750-9-79

**Published:** 2009-09-11

**Authors:** Jana Zimmermann, Isolde Saalbach, Doreen Jahn, Martin Giersberg, Sigrun Haehnel, Julia Wedel, Jeanette Macek, Karen Zoufal, Gerhard Glünder, Dieter Falkenburg, Sergey M Kipriyanov

**Affiliations:** 1 Novoplant GmbH, Am Schwabeplan 1b, 06466 Gatersleben, Germany; 2 Leibniz-Institute of Plant Genetics and Crop Plant Research, Corrensstrasse 3, 06466 Gatersleben, Germany; 3 Clinic for Poultry, University of Veterinary Medicine Hannover, Bünteweg 17, 30559 Hannover, Germany; 4 Current address : Affitech AS, Oslo Research Park, Gaustadalléen 21, 0349 Oslo, Norway

## Abstract

**Background:**

Coccidiosis caused by protozoans of genus *Eimeria *is a chicken parasitic disease of great economical importance. Conventional disease control strategies depend on vaccination and prophylactic use of anticoccidial drugs. Alternative solution to prevent and treat coccidiosis could be provided by passive immunization using orally delivered neutralizing antibodies. We investigated the possibility to mitigate the parasitic infection by feeding poultry with antibody expressing transgenic crop seeds.

**Results:**

Using the phage display antibody library, we generated a panel of anti-*Eimeria *scFv antibody fragments with high sporozoite-neutralizing activity. These antibodies were expressed either transiently in agrobacteria-infiltrated tobacco leaves or stably in seeds of transgenic pea plants. Comparison of the scFv antibodies purified either from tobacco leaves or from the pea seeds demonstrated no difference in their antigen-binding activity and molecular form compositions. Force-feeding experiments demonstrated that oral delivery of flour prepared from the transgenic pea seeds had higher parasite neutralizing activity *in vivo *than the purified antibody fragments isolated from tobacco. The pea seed content was found to protect antibodies against degradation by gastrointestinal proteases (>100-fold gain in stability). *Ad libitum *feeding of chickens demonstrated that the transgenic seeds were well consumed and not shunned. Furthermore, feeding poultry with shred prepared from the antibody expressing pea seeds led to significant mitigation of infection caused both by high and low challenge doses of *Eimeria *oocysts.

**Conclusion:**

The results suggest that our strategy offers a general approach to control parasitic infections in production animals using cost-effective antibody expression in crop seeds affordable for the animal health market.

## Background

Coccidiosis is a diarrheal disease of chickens caused by protozoan parasites of the genus *Eimeria*. It impairs mortality, feed utilization and growth of poultry and causes annual losses of US$ 2.4 billion to the poultry industry worldwide [1]. Conventional disease control strategies depend on vaccination and prophylactic use of anticoccidial drugs. However, resistances against the anticoccidial compounds have already spread and coccidiostats as feed additives will be banned in Europe by the year 2012 [2]. Vaccination strategies with avirulent or attenuated *Eimeria *strains have been routinely used for 50 years, but the large scale production of parasites is relatively laborious and expensive. Limited progress has been achieved towards the development of subunit or recombinant vaccines, the major hurdle being the identification of protective antigens and the delivery (presentation) of the recombinant vaccine to the chicken immune system [1,3,4].

As an alternative strategy, coccidiosis could be prevented by oral delivery of antibodies that inhibit parasite invasion. The complex life cycle of *Eimeria *comprises an exogenous phase in the environment during which the excreted oocysts undergo sporulation. After infection via ingestion of sporulated oocysts, an endogenous phase in the chicken intestine consisting of asexual reproduction (schizogony) and sexual differentiation (gamogony) takes place, followed by fertilization and shedding of unsporulated oocysts [5]. The key step in the disease process in the chicken is the invasion of gut epithelial cells by the parasite. Gut epithelium invasion is accomplished by sporozoites and merozoites as the extracellular invasive stages which represent an attractive target for orally applied inhibitory antibodies. During the last years, numerous monoclonal antibodies against *Eimeria *antigens have been generated, several of them showed an inhibitory effect on sporozoite invasion in the cell culture [6-8]. Furthermore, some monoclonal antibodies, administered intravenously (i.v.) or intraperitoneally (i.p.) for passive immunization, were able to reduce significantly the oocyst output and/or the lesion scores in the chicken gut [9,10].

There are a few major issues precluding use of monoclonal antibodies for preventive and curative passive immunization of the animals against infectious diseases: (i) the costs of antibody production and (ii) the costs of treatment. The treatment costs could be significantly reduced by switching from i.v. or i.p. route of administration to the oral delivery of the feed additives. However, (iii) the natural antibodies *per se *are the serum proteins which easily degrade in the gastrointestinal (GI) tract before they reach their target. All mentioned issues have been addressed in the present study. Using a phage display antibody library, we generated the anti-*Eimeria *antibody fragments with high sporozoite-neutralizing activity. These antibodies were expressed in seeds of transgenic feed pea thus providing a cost-effective antibody production platform affordable for the animal health market. The pea seeds demonstrated the excellent antibody storage properties: dry seeds could be stored at room temperature for long time periods without any loss of antibody activity and they could withstand elevated temperatures during the preparation of feed pellets. Moreover, pea seed content protects antibodies from degradation in the GI tract. The animal experiments demonstrated that *ad libitum *feeding of chickens with fodder comprising the shred prepared from the antibody expressing pea seeds led to a protective and/or curative effect on infection of chickens with *Eimeria *parasite.

## Results

### Generation of a phage display antibody library and selection of *Eimeria*-specific antibodies

To generate recombinant antibodies with *Eimeria *parasite-neutralizing activity, mice were immunized with a mixture containing purified parasite material (oocysts, sporocysts and sporozoites) of five avian *Eimeria *species (*E. tenella*, *E. acervulina*, *E. necatrix*, *E. maxima *and *E. brunetti*). Spleens of mice with the positive immune response were used for mRNA extraction and generation of phage displayed singe-chain Fv (scFv) antibody library of diversity 5.5 × 10^7 ^individual clones. For isolation of antibody fragments binding to *Eimeria *proteins, soluble protein extracts of oocysts, sporozoites and merozoites of *E. tenella *(one of the most economically important *Eimeria *species [1]) were used as complex antigens for panning of the scFv library. After three rounds of panning, amplification, and re-panning, 112 clones expressing scFv were obtained that bound the oocyst extract, 64 clones which bound sporozoite protein, 39 clones that bound membrane proteins from oocysts and sporozoites and 67 clones that bound merozoite proteins (Table 1). Sequence analyses revealed that the selected clones comprise 97 individual scFvs. ELISA demonstrated specific binding of all 97 selected scFv variants to immobilized *Eimeria *antigens with signals significantly higher than the background; 55 scFvs bound to oocyst proteins, 58 to sporozoite proteins and 22 to merozoite proteins. Some scFvs showed specificity to both oocyst and sporozoite or merozoite proteins, thus indicating that the recognized antigens are expressed in different live forms of the parasite (Table 1).

**Table 1:** Antibody variants selected by screening of an anti-*Eimeria *scFv library using complex antigens from *E. tenella*.

**scFv nomenclature**	**Antigen used for screening**	**Number of selected clones**	**Number of unique clones**	**Unique clones showing cross-reactivity with the antigen**		
				
				**Oocyst extract**	**Sporozoite extract**	**Merozoite extract**
AA	Oocyst extract	112	37	37	23	0
AB	Sporozoite extract	64	35	15	35	n.t.
AC	Oocyst and sporozoite membrane proteins	39	3	0	n.t.	0
AD, AE, AF	Merozoite extract	67	22	3	n.t.	22

n.t., not tested.

### Identification of scFv antibodies that inhibit invasion of *Eimeria tenella*

**Figure 1:**
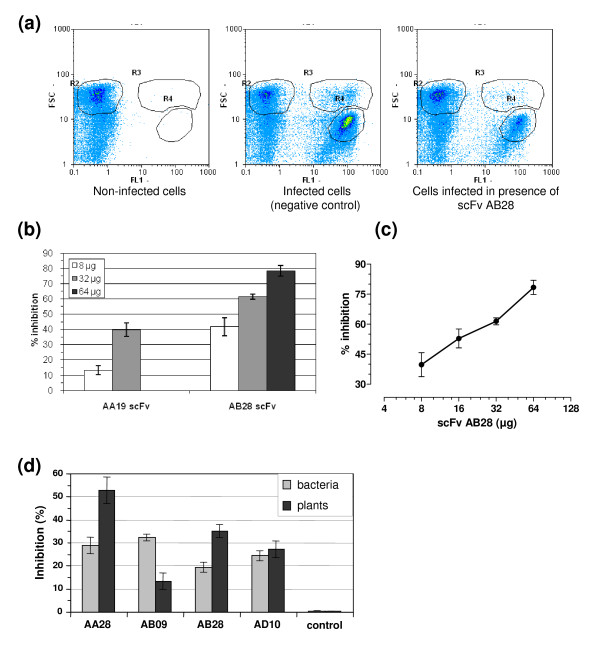
**Inhibition of sporozoite invasion *in vitro *by anti-*Eimeria *scFvs**. (**a**) Analyses of *Eimeria*-infected cells by flow cytometry. Left panel, no infection; middle panel, infected cells (negative control); right panel, infection with sporozoites preincubated with inhibitory scFv AB28 at a concentration of 5.2 μM. In this example, 78% inhibition was observed. (**b**) Comparison of the inhibitory activity of scFvs AA19 and AB28. (**c**) Dose-dependent inhibition of sporozoite invasion *in vitro *by scFv AB28. (**d**) Comparison of anti-sporozoite activity *in vitro *of scFv fragments (10 μg) produced in bacteria or in tobacco leaves. Control, buffer alone.

To identify scFvs that interfere with sporozoite invasion, 28 scFv variants binding to oocyst extracts were selected for further analyses, since they demonstrated distinct sequence variations mainly localized in the complementarity determining regions (CDR). For selection of antibody fragments interfering with cell infection by parasite, the sporozoite invasion assay *in vitro *has been chosen. The method is based on observation that certain cultured mammalian cells, such as Madin-Darby bovine kidney (MDBK) epithelial cells, are susceptible to invasion by sporozoites of *E. tenella *even though these cells are not natural hosts for this parasite. Accordingly, the antibodies specifically binding sporozoite antigens involved in cell invasion can protect the cells from the *Eimeria *infection [11] (Figure 1a). For each scFv, at least two independent invasion inhibition assays were performed. These "activity-screens" were set up to detect any scFv with evidence of inhibitory activity. The threshold that was defined for inhibitory activity was a = 10% reduction of intracellular sporozoites in both independent experiments. This requirement was met by six out of the 28 tested scFvs (Table 2). The scFv inhibition of sporozoite invasion was found to be dose-dependent, as demonstrated by titration of purified antibody fragments (Figure 1b,c).

**Table 2:** Invasion inhibition activity and antigen specificity of the selected scFvs.

**scFv**	**Inhibition of invasion (%)**^**1**^	**Binding to complex antigens**^**2**^			**Putative mol. weight of recognized antigen (kDa)**^**3**^		**Sporozoite surface binding**^**4**^	
		
		**Oocyst** **extract**	**Sporozoite** **extract**	**Merozoite** **extract**	**Reduced**	**Non-reduced**	**FACS**	**IFAT**
AA19	28 ± 14	+	-	-	83-175	83-175	-	-

AA28	36 ± 16	+	-	-	100-150	100-150	-	-

AB09	52 ± 15	+	+	-	n.d.	15-20	+	+

AB21	32 ± 7	+	+	-	n.d.	15-20	+	+

AB28	25 ± 3	+	+	-	Negative	15-20	+	+

AD10	36 ± 12	+	+	-	Negative	15-20	+	+

^1 ^In each experiment, 10 μg bacterially expressed and purified scFv was used. Means and SDs of triplicates are shown.

^2 ^As determined by ELISA.

^3 ^As estimated by comparison with molecular weight markers in Western blot analyses.

^4 ^As determined by IFAT and flow cytometry (FACS).

+, binding; -, no binding; n.d., not determined.

### Analyses of binding properties, antigen- and species-specificity of parasite-neutralizing scFvs

**Figure 2:**
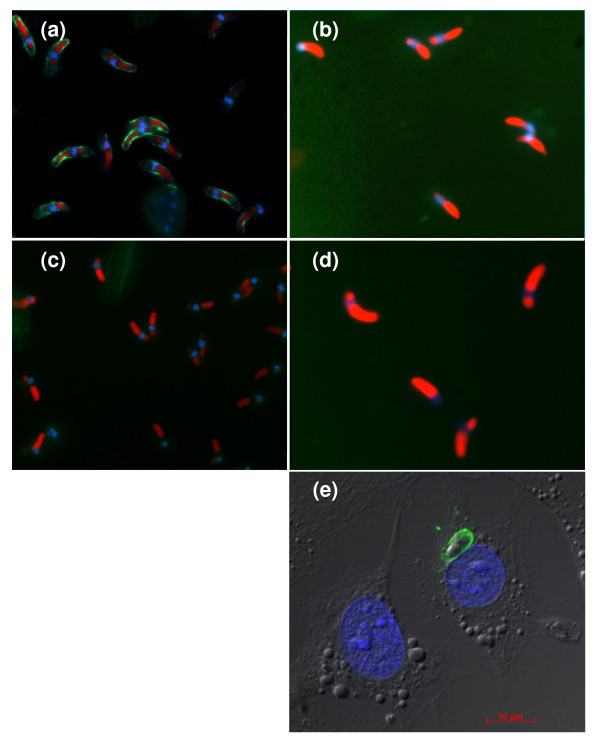
**IFAT analysis demonstrating specific binding of scFv AB28 to sporozoites of *E. tenella *(a) and lack of interaction with sporozoites of *E. acervulina *(b) and *E. brunetti *(d)**. Negative control (binding of irrelevant scFv) is shown in panel (**c**). Blue fluorescence (DAPI), staining of nuclei; red fluorescence (Evans blue), staining of sporozoite refractile bodies and cytoplasm; green fluorescence (FITC), specific antibody staining. In panel (**e**), a fluorescence microscopy image is shown of intracellular sporozoite in infected HepG2 cell. Staining was performed with scFv AB28 followed by anti-c-*myc *MAb and Alexa488-conjugated anti-mouse antibody.

**Figure 3:**
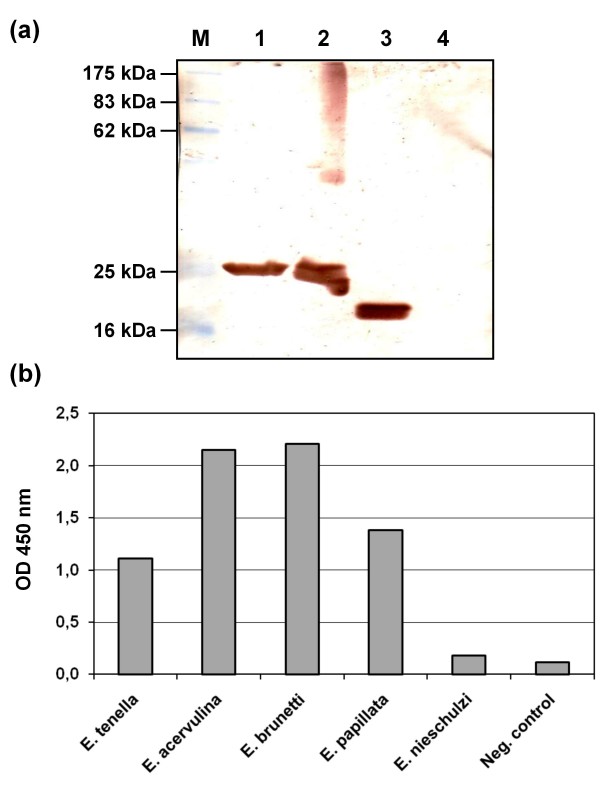
**Analyses of specificity of sporozoite-neutralizing scFvs**. **(a) **Western blot analysis of antigen specificity of scFv AB28. Lanes: M, molecular mass markers (values in kDa are shown on the left); 1,2, produced in *E. coli *25 kDa precursor of a GPI-anchored surface antigen *Et*SAG1 under reducing and non-reducing conditions, respectively; 3,4, oocyst extract of *E. tenella *under non-reducing and reducing conditions, respectively. **(b) **Species specificity of scFv AA28, as determined by ELISA using oocyst extracts from three avian (*E. tenella*, *E. acervulina*, *E. brunetti*) and two rodent (*E. papillata *and *E. nieschulzi*) *Eimeria *species. As a negative control, secondary anti-c-*myc *tag antibody followed by HRP-conjugated goat anti-mouse IgG antibody was used.

A detailed analysis of the antigen specificity of six selected antibody fragments with proven ability to inhibit cell invasion of *E. tenella *sporozoites (AA19, AA28, AB09, AB21, AB28 and AD10) was performed using ELISA, Western blot analysis, flow cytometry and indirect fluorescent antibody test (IFAT). For this purpose, the complex antigens were prepared from different live forms of the *E. tenella *parasites. In addition, a number of known most probable antigen candidates, such as immunodominant surface antigen *Et*SAG1 (TA4) [12]; microneme proteins *Et*Mic1 [13], *Et*Mic2 [14], *Et*Mic3 [11] and *Et*Mic5 [15]; Eimepsin (aspartyl proteinase) [16]; merozoite antigen MZP 5-7 [17] and a 19 kDa antigen 3-1E (potential immunostimulator) [18] were generated and expressed as recombinant proteins. Since the *Et*SAG1 antigen as isolated from the parasite is composed of a 17 kDa polypeptide (large subunit) and a 8 kDa polypeptide (small subunit) linked by a disulfide bridge [12], recombinant *Et*SAG1 protein was produced in bacteria as a 25 kDa precursor without a signal peptide and a glycosylphosphatidylinositol (GPI) anchor. The results of analyses are summarized in Tables 2 and 3. Out of six tested, four scFv candidates (AB09, AB21, AB28 and AD10) demonstrated clear staining of the surface of *E. tenella *sporozoites (Table 2; Figure 2). Moreover, these four variants showed specific binding to the recombinant *Et*SAG1 precursor (Table 3; Figure 3a). SDS-PAGE and Western blot analyses revealed that all *Et*SAG1-specific scFvs (AB09, AB21, AB28 and AD10) recognized the large 17 kDa subunit of *Et*SAG1 in oocyst and sporozoite extracts of *E. tenella *under non-reducing conditions (Table 2; Figure 3a). In contrast, no antibody binding was detected when the oocyst extract was reduced (Table 2; Figure 3a). Interestingly, the *Et*SAG1-specific scFvs retained binding to the 25 kDa precursor also under reducing conditions.

**Table 3:** Antigen and species specificity of the selected scFvs with anti-sporozoite invasion activity, as determined by ELISA (E) and Western blot analyses (W).

**scFv**	**Recombinant antigen**							
	
	***Et*SAG1**	***Et*Mic1**	***Et*Mic2**	***Et*Mic3**	***Et*Mic5**	**Eimepsin**	**MZP 5-7**	**19 kDa Ag**
AA19	-(E)	-(W)	-(E)	-(W)	-(W)	-(W)	-(E)	-(E)

AA28	-(E)	-(W)	-(E)	-(W)	-(W)	-(W)	-(E)	-(E)

AB09	+(E, W)	-(W)	-(E)	-(W)	-(W)	-(W)	-(E)	-(E)

AB21	+(E)	n.t.	-(E)	n.t.	n.t.	n.t.	-(E)	-(E)

AB28	+(E, W)	-(W)	-(E)	-(W)	-(W)	-(W)	-(E)	-(E)

AD10	+(E, W)	n.t.	n.t.	n.t.	n.t.	n.t.	n.t.	-(E)

								

**scFv**	***Eimeria *species**							
	
	***E. tenella***		***E. acervulina***	***E. brunetti***		***E. papillata***		***E. nieschulzi***

AA19	+(E, W)		+(E, W)	+(E)		+(E)		-(E)

AA28	+(E, W)		+(E, W)	+(E, W)		+(E)		-(E)

AB09	+(E, W)		-(E, W)	-(E)		-(E)		-(E)

AB21	+(E, W)		-(E, W)	-(E)		-(E)		-(E)

AB28	+(E, W)		-(E, W)	-(E, W)		-(E)		-(E)

AD10	+(E, W)		-(E, W)	-(E)		-(E)		-(E)

+, binding; -, no binding; n.t., not tested.

For analysis of cross-reactivity of the scFv fragments selected for inhibition of invasion of *E. tenella *sporozoites with other *Eimeria *species, the complex soluble antigens were prepared from sporulated oocysts of three avian *Eimeria *species (*E. tenella*, *E. acervulina *and *E. brunetti*) and two rodent *Eimeria *species (*E. nieschulzi *and *E. papillata*). As expected, all *Et*SAG1-specific scFvs showed reactivity only with *E. tenella *(Table 3). The scFv variants AB09, AB21, AB28 and AD10 did not interact with the protein preparations from other species, thus indicating that they recognize *Et*SAG1 epitopes which are unique for *E. tenella*. In contrast, the scFvs AA19 and AA28 demonstrated fairly broad species cross-reactivity (Table 3; Figure 3b). However, these variants neither bound to sporozoite and merozoite extracts nor interacted with the surface of sporozoites (Table 2).

### Oral application of tobacco expressed antibody fragments in chickens infected with *Eimeria*

**Figure 4:**
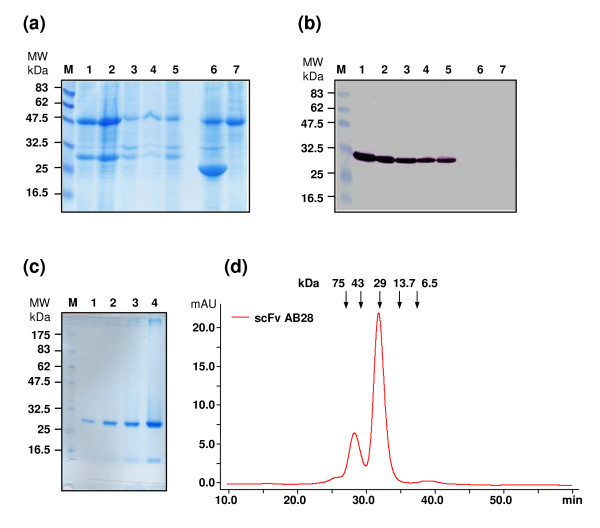
**Analysis of scFv AB28 expression in tobacco leaves**. **(a, b) **Reducing 12% SDS-PAGE analysis of leaf discs: Coomassie staining **(a) **and Western blot with detection using anti-His antibody **(b)**. Lanes: M, molecular mass markers (values in kDa are shown on the left); 1-5, leaf materials after expression of scFv AB09, AB28, AD10, AA28 and AB21, respectively; 6,7, negative controls (tobacco expressing GFP and wt plants, respectively). **(c,d) **Characterization of tobacco expressed scFv AB28 after purification. **(c) **Coomassie stained 12% SDS-PAA gel. Lanes: M, molecular mass markers; 1-4, scFv samples (1.3, 2.6, 5.2 and 13 μg, respectively). **(d) **Analysis of scFv AB28 molecular forms by size-exclusion FPLC on a calibrated Superdex 200 column. The positions of the molecular weight markers are indicated.

Oral application of antibodies for prevention of *Eimeria *infections in poultry would only be feasible if cheap sources of large amounts of recombinant protein were available. Production in plants provides an economically attractive source of recombinant antibodies [19]. Therefore, the recombinant anti-*Eimeria *antibody fragments were first expressed in leaves of the tobacco plant *Nicotiana benthamiana *as a quick source of large amounts of plant-made recombinant protein. Typically, one-step purification yielded 20, 100, 90, 400 and 60 mg of recombinant scFv variants AA28, AB09, AB21, AB28 and AD10, respectively, with purity above 90% from 1 kg wet weight of infiltrated tobacco leaves (Figure 4). Comparison of the anti-sporozoite activity *in vitro *of the antibody fragments isolated either from tobacco or from *E. coli *demonstrated superior properties of the plant-produced material for most of the tested anti-*Eimeria *scFvs (Figure 1d).

**Figure 5:**
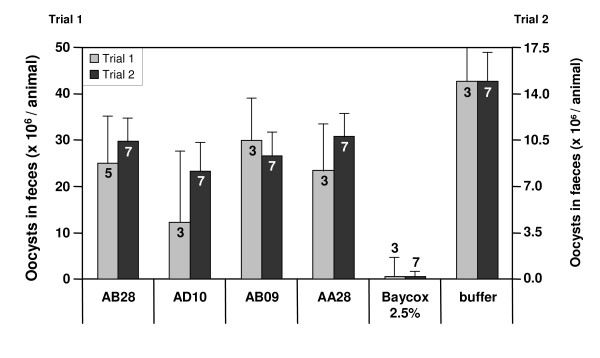
**Evaluation of activity *in vivo *of orally applied anti-*Eimeria *antibodies**. Infection outcomes were assessed by quantifying the amounts of *Eimeria *oocysts in feces of the infected chickens. Birds in the treatment groups received 1 mg antibody isolated from tobacco leaves in 1 ml PBS a day via gavage. The negative treatment group received buffer alone. Animals of the positive control group received 1 ml of a 2.5% solution of the anti-coccidial drug Baycox^® ^(Bayer HealthCare) in water. The results of two independent studies are presented. Mean values and SDs for each treatment group are plotted. The numbers of animals in each chicken cohort are indicated on the bars.

Animal studies were performed to assess whether the scFv-mediated inhibition of sporozoite invasion observed *in vitro *also prevents or mitigates *Eimeria *infections in chickens upon oral delivery of the scFvs. For these experiments, we used purified antibody fragments produced and isolated from the tobacco leaves. The chickens were orally infected with a single dose of *E. tenella *oocysts (500 oocysts per bird). One day before infection, force-feeding was started with the scFv fragments and continued for 9 days. Birds in the treatment group received 1 mg antibody isolated from the tobacco leaves in 1 ml PBS a day via gavage. The results of two independent animal trials performed in the same way are summarized in Figure 5. The untreated control groups showed oocyst shedding starting on day 6 after challenge and increasing throughout day 7. At the same time points, treatment with the commercial anti-coccidian drug Baycox^® ^reduced oocyst shedding to marginal levels. Animals that received scFvs experienced a partial (35-60%) reduction in oocyst numbers in comparison with the untreated controls; however, there was no significant difference. The individual data sets of scFv-fed groups showed a fairly high variability between the experiments. Nevertheless, all tested scFv variants demonstrated a somewhat positive effect on oocyst shedding with the highest inhibition rate of 60% observed for the group treated with a scFv AD10.

### Pea plant transformation and expression of scFvs in pea seeds

Although the transient expression of heterologous proteins in leaves of *N. benthamiana *allows relatively quick production of reasonable quantities of the scFv fragments for proof-of-concept (POC) animal studies, application of antibodies as fodder additives in routine poultry feeding requires more efficient and cost-effective production systems. Therefore, stable expression of single-chain antibody fragments was established in the fodder pea variety "Eiffel". For generation of the transgenic pea lines, the genes of scFv fragments of two different specificities (anti-*Et*SAG1 scFv AB28 specific for *E. tenella *and scFv AA28 with the broadest cross-reactivity to *Eimeria *species) were selected. The transgenic pea lines have been generated which express scFvs under the control of a seed-specific promoter. The results of characterization of AB28-expressing pea lines are summarized in Additional files 1, 2, 3 &4. Similar analyses were performed for the AA28-transgenic pea lines. The tests demonstrated that scFv expression was restricted to seeds, where the scFv protein could unambiguously be detected by Western blot analysis (Additional file 1). For estimation of the expression levels, the scFv antibody fragments were isolated from the pea seeds and purified. Three independent measurements yielded 1.76 ± 0.41 mg extractable functional scFv AB28 per 1 g dry seed weight. Furthermore, the scFv expression levels stayed the same over a number of following homozygous pea generations (1.66, 1.92, 1.64 and 1.90 mg scFv per 1 g dry seeds of generations F4, F5, F6 and F7, respectively). Head-to-head comparison of scFv AB28 preparations isolated either from the tobacco leaves (n = 5) or from the pea seeds (n = 3) demonstrated nearly identical molecular form compositions in different preparations. The scFv material from both tobacco leaves and pea seeds was mostly monomeric (73.10 ± 6.27% and 76.65 ± 13.22% for the tobacco and pea material, respectively) with presence of a dimeric (diabody [20]) fraction (25.68 ± 6.08% and 20.80 ± 9.62%, for the tobacco and pea material, respectively) and some minor quantities of higher molecular forms, most probably tetramers (1.17 ± 0.44% and 2.5 ± 0.35%, for the tobacco and pea material, respectively) (Figure 4d and Additional file 5). Accordingly, the scFv preparations from the tobacco leaves and pea seeds demonstrated very close similarity (*P *= 0.192) in their antigen-binding activities, with the calculated K_D _values of 3.53 ± 1.24 nM (n = 3) and 2.60 ± 0.73 nM (n = 5), respectively, as determined by ELISA (Additional file 5).

### Analysis of the proteolytic and pH stability *in vitro*

**Figure 6:**
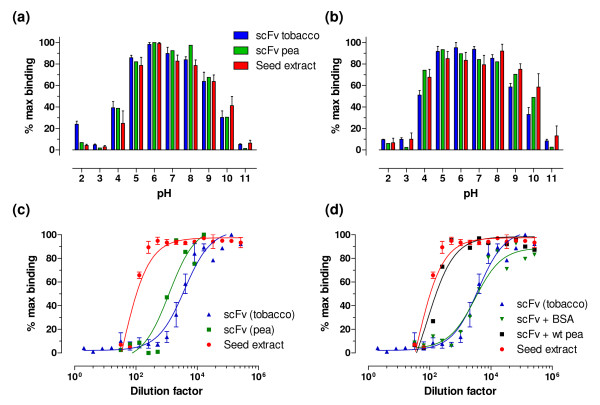
**Analyses of pH and proteolytic stability of scFv AB28 preparations purified either from tobacco leaves or pea seeds and of the AB28-containing pea seed extract**. **(a, b) **pH influence on formation ("binding") and disruption ("dissociation") of the antibody-antigen complexes formed by different AB28-preparations (**a **and **b**, respectively). **(c, d) **Loss of antigen-binding activity of scFv AB28 preparations as a result of degradation by intestinal proteases. **(c) **Comparison of purified scFv AB28 isolated either from tobacco leaves or from pea seeds as well as of crude protein extract from the scFv-expressing seeds (seed extract). **(d) **Effect of irrelevant protein (BSA) or wt pea seed extract on proteolytic stability of scFv AB28 (tobacco) in chicken intestinal fluid. For comparison, a degradation curve for AB28-transgenic seed extract is shown. The residual antigen-binding activity was determined by ELISA using the plates coated with the oocyst extract of *E. tenella *and plotted against the chicken intestinal fluid dilutions.

For an effective prevention of parasite invasion into the intestinal epithelium of chickens, the antibody fragments must survive the harsh conditions of the GI tract, in particular, high protease content and acidic pH, for a longer time. It is known that avians possess the same basic structures for nutrient extraction as other vertebrates but also exhibit specific features within their GI tract. These include a crop for storage of feed, a proventriculus (simple stomach), a gizzard and paired caeca. The pH values of specific sections of the chicken GI tract are the following: crop 4.5, proventriculus 4.4, gizzard 2.6, duodenum 5.7 to 6.0, jejunum 5.8, ileum 6.3, colon 6.3, caeca 5.7, and bile 5.9 [21]. We, therefore, performed comparative analyses of influence of the expression system on the pH and proteolytic stability of the antibody fragments using as an example the *Et*SAG1-specific scFv AB28. First, we tested the influence of different pH conditions separately on formation of the antibody-antigen complexes ("binding") as well as on dissociation of already preformed antigen-antibody contacts ("dissociation"). The purified scFv AB28 preparations isolated either from tobacco leaves or from the transgenic pea seeds were compared. In addition, crude protein extract prepared from AB28-expressing pea seeds was included into the assay. The results presented in Figure 6a,b demonstrate a fairly comparable effect of pH on all three antibody preparations. As expected from the general considerations, the marginal pH values had a more pronounced negative effect on the formation of the antigen-antibody complexes than on dissociation of the already bound antibody. In general, the found pH effect on formation and dissociation of the antibody (Ab)/antigen (Ag) complexes rather reflects the stability of the Ab/Ag interface partly formed by the electrostatic interactions and hydrogen bonds between the charged and polar amino acid side chains than the folding/unfolding stability of the antibody molecules themselves. In conclusion, all tested scFv AB28 preparations retained 70-100% of their maximal antigen-binding activity under the pH conditions prevalent in the caeca where the *E. tenella *sporozoite invasion takes place [22].

To simulate the protease-rich conditions of the small intestine, activity and stability of different scFv AB28 preparations were tested in presence of enteric proteases present in the intestinal fluid (chyme) isolated from the small intestine of chickens. Comparison of purified scFv AB28 preparations isolated either from the tobacco leaves or from the pea seeds demonstrated a three-fold higher resistance of pea-expressed material against proteolytic degradation in the small intestine (Figure 6c; Table 4). The scFv material present in crude pea seed extract appeared even more stable, 40-fold more resistant than scFv purified from pea seeds and 100-fold more stable than scFv from tobacco. This finding indicates a protective anti-protease effect of the pea seed content. To analyze whether this protective effect is seed-specific or just caused by the high protein content of the pea flour, we compared proteolytic stability of the tobacco-produced scFv AB28 in the presence of either wild-type (wt) pea seed extract or irrelevant protein, such as bovine serum albumin (BSA). The final BSA concentration was 15% (w/v), as adjusted according to the measured protein content of the pea seed extract. The results presented in Figure 6d and Table 4 indicate that presence of excess of irrelevant protein had no effect on scFv degradation in chicken intestinal fluid and that the stabilizing effect could be attributed exclusively to the content of the pea seed extract (45-fold gain in stability). The tobacco-made scFv AB28 premixed with the wt pea seed extract proved nearly as stable as an extract prepared from the AB28-expressing transgenic pea seeds (Figure 6d).

**Table 4:** Effect of expression system and additives on proteolytic stability of scFv AB28.

**scFv preparation**	***ED***_**50**_
scFv (tobacco)	3,786.00

scFv (pea)	1,209.00

scFv (tobacco) + 15% BSA	3,053.00

scFv (tobacco) + wt pea seed extract	83.24

AB28-pea seed extract	33.47

*ED*_50_, effective dilution of chicken intestinal fluid leading to 50% loss of activity.

### Reduction of parasite shedding upon force-feeding of chickens with pea seed flour prepared from scFv-expressing seeds

To test whether pea seeds expressing protective scFvs can avert infection with *E. tenella*, the flour prepared from the AB28-expressing seeds was used for treatment of chickens infected with *E. tenella *oocysts. For comparison, expressed in tobacco and purified scFv AB28 and an irrelevant scFv BA11 raised against enterotoxigenic *Escherichia coli *(ETEC) as well as BA11-containing pea seed flour were used. The study was designed to mimic the normal housing conditions, such as keeping birds in clean stables where only a few oocysts could be present which survived the disinfection procedure. A preliminary dose finding study demonstrated that infection with a dose as low as 7-15 oocysts per chicken results in measurable oocyst shedding. In addition, lowering the infection dosages led to a significant reduction of deviation between the individual animals. Therefore, the animals were infected with manually prepared individual doses of 20 ± 1 oocysts. The chickens were force-fed three times daily either with 1 mg in total of scFvs purified from the tobacco leaves or with the flour prepared from the scFv-expressing pea seeds. In latter case, the maximal delivered dose of functional scFv was estimated to be 0.5 mg per bird a day (see Methods).

**Figure 7:**
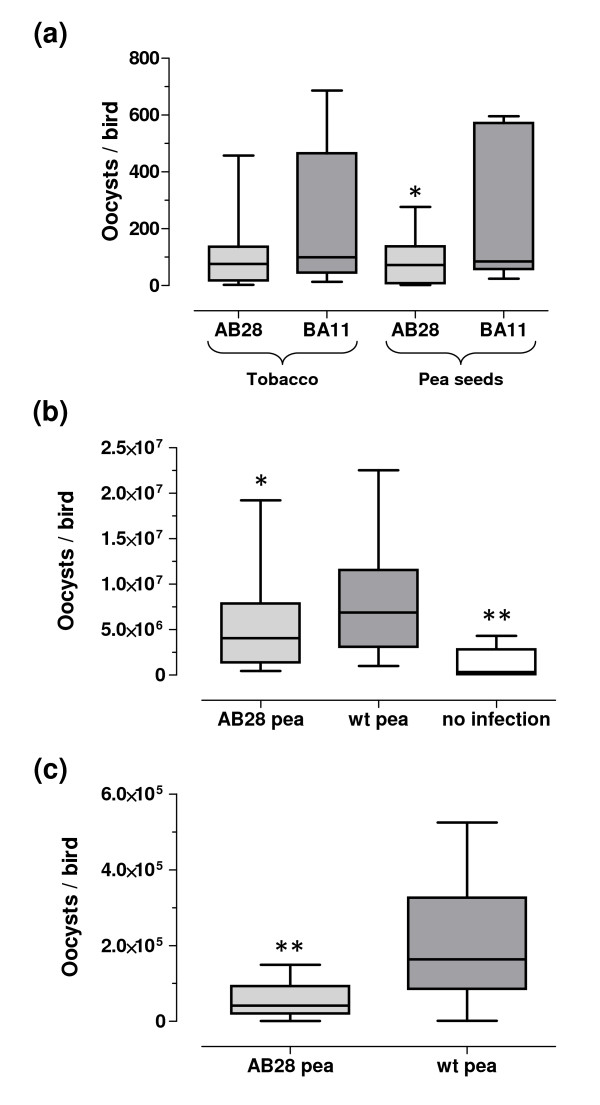
**Evaluation of anti-*Eimeria *activity *in vivo *of fodder containing antibody-expressing pea seed flour or shred**. **(a) **Analysis of activity *in vivo *of orally applied antibody-containing pea seed flour (force-feeding). The animals were infected with manually prepared individual doses of 20 ± 1 oocysts. Two evaluation groups (n = 11) were treated with the material containing anti-*Eimeria *scFv AB28, one group received pea flour from seeds expressing AB28 and the other group was treated with the antibody fragment isolated from the tobacco leaves. As positive controls of infection, two animal groups (11 birds in each) were treated with the same quantities of material comprising the irrelevant BA11 antibody, either in form of pea flour or as soluble antibody isolated from the tobacco leaves. The negative control group comprised five birds to who heat inactivated oocysts were given. The oocyst counts were determined in individual feces and each sample was counted six times by two different persons. The infection outcomes were assessed by quantifying the amount of *Eimeria *oocysts in feces of infected chickens at days 7 and 8 p.i. (days of maximal oocyst release). **(b) **High infection dose model (*ad libitum *feeding). Three treatment cohorts were evaluated: group 1 (n = 30) received fodder with 10% AB28-pea shred; group 2 (n = 25), positive control for infection (infected, not treated); group 3 (n = 5), negative control of infection (non-infected, not treated). The birds in groups 2 and 3 were fed with the fodder containing 10% wt pea. Infection outcomes were assessed by quantifying the amounts of the *Eimeria *oocysts in caeca of infected chickens at day 7 p.i. **(c) **Low infection dose model (*ad libitum *feeding). Infection outcomes were assessed by quantifying the amounts of the *Eimeria *oocysts in caeca of infected chickens at day 7 p.i. Due to the relatively low infection rate, only the oocyst shedding animals were considered, i.e. 17 birds from the group 1 (AB28) and 13 animals from the group 2.

The results of the study are shown in Figure 7a. The average oocyst shedding was 1.5-fold lower in birds treated with the expressed in tobacco and purified scFv against *Eimeria *(AB28) in comparison to birds that were treated with the same amount of a purified irrelevant scFv (BA11), although the difference was not significant (*P *= 0.116). However, treatment with AB28-containing pea seed flour resulted in a significant 2.5-fold lower average oocyst shedding than in the animal group receiving BA11 pea flour (*P *= 0.033). For groups treated with the pea material, the anti-*Eimeria *scFv antibody AB28 showed an infection inhibition of 60.5%. For groups treated with the purified material, essentially lower inhibition (34.7%) was observed.

### Mitigation of *Eimeria tenella *infection by application of scFv-containing pea seeds in chicken feed

For demonstration of the potential of fodder containing AB28-pea shred in prevention or mitigation of the coccidian infection caused by *E. tenella*, the controlled battery experiments were performed under normal *ad libitum *feeding conditions. To determine whether the transgenic pea shred is accepted as a part of the feed and readily taken up by chickens as well as to assess the digestibility of the pea shred, a preliminary feeding study was performed using a fodder containing 10% pea shred derived from the pea seeds expressing irrelevant anti-ETEC scFv antibody BA11. The results shown in Additional file 6 demonstrated no difference in weight gain and feed consumption between the BA11-fed chickens and the animals of the control group. The transgenic pea shred was well consumed by broiler chickens and was not shunned (Additional file 6). ELISA analyses demonstrated absence of the BA11-activity in extracts from the fecal material, thus indicating that the recombinant antibody present in transgenic pea seeds was completely digested (data not shown).

In our feasibility studies, we evaluated high- and low-dose infection models. The high dose model was designed to get appropriate lesion scores for differentiation of infections. However, taking into account that the lesion score analysis is not always a reliable method to measure the efficacy of anti-coccidials [23], the reduction of oocyst counts in caeca was determined as an endpoint of the study. Broiler chickens were infected orally with individual doses of 2,500 freshly prepared oocysts of *E. tenella*. Three animal cohorts were formed; in two groups birds were infected and fed with the fodder comprising 10% pea shred derived either from AB28-expressing seeds (treatment group) or from the wild-type pea (positive control of infection). Adapted from the preliminary feeding experiment, the average consumption of the scFv antibody by chickens of the treatment group was estimated as 35.63 ± 1.87 mg antibody/kg body weight × day (Additional file 7). The negative control group (control of spontaneous infection) comprised birds which were not infected and received conventional fodder. At the end of experiment, the lesion scores and the oocyst counts were determined in caeca of sacrificed birds. The results of the study demonstrated that there was no significant difference in the body weight gains (*P *= 0.068) and severity of the lesions (*P *= 0.205) between the infected groups. However, less animals in the AB28-treated group had blood in feces (40% vs. 44% in the positive control group) and a higher proportion of the AB28-treated animals had lightweight lesions (50% AB28-pea fed animals had lesion scores 0 and 1 vs. 40% in the positive control group). Accordingly, only 26% of the AB-28 treated animal had lesion score 3 versus 36% in the positive control group. Although the mentioned differences were not statistically significant, the observed trend was confirmed when the oocyst counts from caeca were compared (Figure 7b). The average oocyst counts were significantly lower in birds fed with the AB28-expressing pea seeds than in animals receiving conventional fodder (*P *= 0.038). Feeding with AB28-pea led to reduction of the oocyst counts in caeca to 65.9% of the numbers found in the positive control group. Therefore, the inhibition of invasion caused by the AB28-transgenic fodder could be calculated as 34.1%.

The low-dose infection model was employed to mimic the real situation in barns for rearing commercial broilers where the chickens are spontaneously infected with relatively low dosages of *Eimeria *oocysts. The feeding experiment was performed under nearly the same conditions as the high-dose infection experiment, only the infection dose has changed. In this study, the chickens were infected with the ten-fold lower oocyst doses, i.e. 250 oocysts per bird. Due to the low oocyst doses, the vast majority of infected chickens had reduced lesion scores 0 and 1 (100% and 96% in AB28-treated and in positive control groups, respectively). The animal cohort fed with AB28-pea seeds demonstrated significantly lower oocyst counts in caeca (*P *= 0.032). However, about one third of animals in both infected groups stayed oocyst-free most probably due to the innate immunity against the pathogen. Therefore, the effect of modified feed was also separately assessed only for the oocyst-shedders in each group. The results presented in Figure 7c demonstrate very significant reduction of caecal oocyst counts in the birds fed with the AB28-containing fodder (*P *= 0.002). In summary, feeding with AB28-pea seeds led to a reduction of the oocyst counts in caeca to a level of 29.2% from the counts found in the positive control group. Therefore, the inhibition of invasion caused by the scFv AB28 pea seed material could be calculated as 70.8%.

## Discussion

In view of the spread of microbial resistance to antibiotics and the emergence of new pathogens, passive immunization by recombinant antibodies is considered as one of the most promising alternatives to combat infectious diseases [24,25]. Although the market for therapeutic monoclonal antibody-based products is one of the fastest growing segments in the biopharmaceuticals industry, this market is heavily focused on oncology, autoimmune and inflammatory diseases. The role of antibodies for mitigation and therapy of infections is only slowly emerging, but is impeded by the high Cost of Goods (COG). High COG also prevents successful introduction of antibodies into the animal health market. Plant-based production provides a solution to these cost problems. In addition, plants can provide an adequate system for oral delivery of recombinant biomolecules as part of the diet. Infrastructure and costs for downstream processing can thus be avoided, as well as the production losses which are often significant. Although expression of recombinant antibodies and antibody fragments in plants ("plantibodies") has been demonstrated two decades ago [26], their introduction into the health and veterinary markets is still years away. In our knowledge, the present report is a first demonstration of feasibility to control poultry infections by feeding animals using a fodder supplemented with the antibody producing transgenic seeds.

The first objective of this study was generation of recombinant neutralizing antibodies against the economically important protozoan parasites of genus *Eimeria*. An immune library was constructed by phage display and panned against the complex antigens prepared from the different live forms of *E. tenella*. Since the orally delivered antibodies cannot rely on effector functions of circulating immunoglobulins such as Fc-receptor mediated recruitment of the immune killer cells (ADCC) or complement fixation (CDC) inside the GI tract, the only possible mechanism of action would be prevention of the parasite entry into the gut epithelium. Therefore in contrast to the previously described approaches [27,28], we introduced screening of binders for the parasite neutralizing activity early into the selection process. This strategy resulted in identification of six candidates; four of them appeared to be specific for *Et*SAG1 (TA4) sporozoite antigen of *E. tenella*. *Et*SAG1 has been found previously by us and others to be a molecular target for a number of sporozoite-neutralizing antibodies [12,29,30] and seems to be closely involved into initiation of the infection process [30]. The antigen specificities of two neutralizing non-*Et*SAG1 binders and the mechanism of their infection-blocking activity are still unknown, since both these variants bound neither sporozoites nor merozoites.

The second goal of the study was testing the suitability of orally applied antibody molecules for controlling the *Eimeria *infection. To make the product compatible with the already existing cost structures in the animal health market, the cost efficient plant production systems have been utilized. First, the selected antibody fragments were transiently expressed in agrobacteria-infiltrated tobacco leaves and purified. Besides the higher production yields, the tobacco-derived antibody fragments demonstrated superior biological activity *in vitro *in comparison with the counterparts expressed and purified from bacteria. Furthermore, the relatively small quantities of tobacco-made scFv fragments (1 mg per chicken a day, 8 day treatment) were able to cause partial reduction of *Eimeria *oocyst shedding by the infected animals.

Although the transient and stable expression in tobacco leaves allows the cost-reasonable industry-scale production of heterologous proteins [31,32], it requires fairly intense product purification strategies, to remove at least the toxic alkaloids from the tobacco extracts. In contrast, expression of the therapeutic proteins in crop seeds has a great advantage for oral administration since only minimal treatment is necessary to use seeds as the livestock fodder. The other advantages of crop plants include easy upscaling through field cultivation, established harvesting and processing technologies, as well as easy seed storage and distribution. Seeds are natural storage organs, with the optimal biochemical environment for the accumulation of large protein amounts. In this form, proteins can be transported from farm to the production factories without any loss of stability. Moreover, feasibility of high-level production and long-term storage of antibodies in seeds has already been demonstrated [33,34]. In our work, we have selected feed pea as a production plant on the basis of the following considerations. Pea is an established field crop with available infrastructure and tools for large scale planting, weeding, harvesting, storage and processing. Furthermore, the pea seeds are an established feed/food ingredient with high protein content (up to 40% [35]). Pea has a history of successful heterologous expression studies including field trials (own unpublished data) and has excellent safety features. The safety aspects include strict self-pollination; lack of outcrossing; no dispersal of seeds by wind and, therefore, very low number of volunteers; lack of toxic substances in seeds. The eaten seeds get digested and thus are not able to germinate; the pea plants do not survive frost. In addition, the pea varieties are approved as crop plants for livestock fodder. Due to the different sugar and glycopeptides contents, the feed pea has different taste and texture than the food pea, thus allowing easy discrimination to prevent potential entering of GMO into the food chain. In addition, using seed-specific promoter makes expression of the transgenic protein nearly totally restricted to the seeds [36]. The safety features are very important in view of tight regulatory control and surveillance of genetically modified (GM) plants [37].

We have, therefore, generated transgenic pea plants expressing two most promising scFv candidates in seeds. The highest producer, a homozygous pea line expressing anti-*Et*SAG1 scFv AB28 at yields of 1.5-2 g extractable functional antibody per 1 kg dry seeds, was taken for the detailed analyses and feasibility studies in animals. Comparison of the scFv fragments isolated from the pea seeds with the counterparts expressed in tobacco leaves (a plant expression system most often used for production of antibodies and their fragments [31,34]) demonstrated no difference in the molecular form composition, antigen binding activity and pH stability, but a somewhat higher resistance of the pea seed derived material against intestinal proteases. Comparative analysis of the proteolytic resistance revealed a key protective role of the pea seed extract in scFv stability. The stabilizing effect can be attributed to the well-known presence of the proteinase inhibitors in legume seeds [38]. The trypsin/chymotrypsin inhibitors found in pea seeds mainly belong to the Bowman-Birk protease inhibitor (BBI) family. BBIs are stable at cooking temperatures and also towards acidic pH values in the digestive systems of humans and animals, most probably due to their large number of disulfide bonds (seven bonds out of about 70 amino acid residues) and the polar interactions between the sub-domains. The potential applications of BBIs have recently attracted much attention. BBIs are already used to defend against insects in transgenic plants, and they also have prospects in the prevention of cancer, Dengue fever, and inflammatory and allergic disorders (for review see [39,40]). These findings provide an additional argument for choosing the legume seeds as the production system suitable for passive immunization.

The controlled feeding experiments demonstrated statistically significant positive effect of using antibody-expressing transgenic pea seeds on mitigation of coccidial infection. In first instance, force-feeding with the pea flour suspension appeared to be more efficient than the oral administration of twice larger amounts of purified antibody fragments. The found effect was additionally confirmed by *ad libitum *feeding using both high- and low-dose infection models. The challenge doses of *E. tenella *oocysts that were used in the first model (2.5 × 10^3^) may be considerably greater than the levels that commercial birds are likely to be exposed to in some production facilities. Nevertheless, the observed endpoint of the study (reduction of oocyst counts is caeca) was still significant. Much better results were obtained by using the ten-fold lower challenge oocyst doses. Therefore, the transgenic pea seeds may be useful for protection against coccidiosis in poultry raised under normal field conditions, as we demonstrated using the low-dose infection model.

Although the results presented here are rather preliminary, they provide evidence that the recombinant antibodies produced in pea seeds have capacity to reduce the infectious loads in animals following oral administration. Product-specific issues such as antibody stability in the gut, parasite neutralizing potency or even direct killing activity have to be addressed, as well as technical issues (increasing the production yields and shortening time for GM plant generation) and commercial applicability. For example, in the present study we used the scFv variants selected from the phage display immune library without any further optimization. Affinity maturation of the scFv candidates and improving their proteolytic stability and folding efficacy in plants using either random or rational approaches [41] may further contribute to the success of the described passive vaccination strategy. The antibody stability against intestinal proteases can also be enhanced by conversion of scFv into a bivalent single-chain antibody format without compromising the expression yields in plants that is normally observed for heterooligomeric full-length antibodies of IgG and IgA classes or Fab fragments (Giersberg *et al*., manuscript in preparation). Alternatively, well expressed and proteolytically stable binding proteins selected from the non-antibody scaffold libraries can be used (for review see [42]). In addition, further enhancement of anti-parasite potency can be achieved, for example, by grafting the killing antimicrobial peptides into the antibody CDR loops not involved into the antigen binding [43].

## Conclusion

Taken together, this study demonstrated for the first time beneficial effect of using antibody expressing pea seeds for passive protection against avian coccidiosis in newly hatched birds. Compared with methods of active vaccination either by live parasites (wild-type and/or attenuated strains) or using recombinant subunit or DNA vaccines [1], the passive immunization strategy described here is an easy and non-invasive method to use in commercial settings with the comparatively low cost of production utilizing current agriculture technologies and with the ability to be used in combination with other anti-parasitic agents.

## Methods

### Immunization of mice and generation of a phage display antibody library

For library generation, six BALB/c mice were immunized by injecting 100 μl of a mixture containing 1.0 to 3.9 × 10^6 ^purified parasites (oocysts, sporocysts and sporozoites) of five *Eimeria *strains (*E. tenella*, *E. acervulina*, *E. necatrix*, *E. maxima *and *E. brunetti*). For immunization, the *Eimeria *oocysts were sporulated and used for isolation of viable sporozoites as described by Raether *et al*. [44]. The protein extracts from sporulated oocysts and sporozoites were prepared by vortexing the parasite stages with glass beads in PBS containing 1 mM PMSF, followed by six freeze-thaw cycles for parasite disruption and sonication on ice. Soluble extracts were obtained by additional centrifugation for 10 min at 13,000 *g*. Three immunizations were applied on day 1, day 21 and day 41, respectively. Successful immunization was monitored by performing IFAT tests with immobilized *E. tenella *sporozoites. RNA was isolated from spleens of the mice (RNAeasy Midi-Kit, Qiagen) and polyA^+ ^RNA purified (Oligotex-mRNA-Mini-Kit, Qiagen). First strand cDNA was obtained using the SuperScript First-Strand Synthesis System for RT-PCR from Gibco BRL. PCR amplification of the V_H _and V_L _regions using minimal primers Bi3f/Bi4 for V_H _and Bi5c/Bi8b for V_L _[45] was used as quality-control for the cDNA. For the construction of the phage-display library, cDNA encoding the V_H _and V_L _domains was generated by PCR using a described mouse immunoglobulin primer set [46]. As a vector for phage surface display, a phagemid pEXHAM1 [47] was used which was derived from pSEX81 [48] by inserting a DNA sequence encoding a tag that consists of six histidine residues (His6-tag), an amber stop codon (Am), and a c-*myc *epitope between the scFv and the M13 g3p gene. The amplified V_H _genes were cloned as *Nco*I-*Hind*III fragments to substitute a V_H _gene of a dummy scFv containing a YOL-linker [49] in the pEXHAM1 vector. The ligated phagemids were used for transformation of *E. coli *XL1-Blue cells (Stratagene) to generate a V_H _sublibrary of 10^7 ^individual clones. The produced DNA was used for cloning the V_L _gene repertoire as *Mlu*I-*Not*I DNA fragments. The overall achieved scFv library complexity was 5.5 × 10^7 ^individual clones.

### Screening of the phage-display library and selection of *Eimeria*-specific antibodies

The antigen immobilization and library screening was performed according to published protocols [45,46]. For isolation of scFvs that bind to *Eimeria *proteins, soluble protein extracts of *E. tenella *oocysts, sporozoites and merozoites were used as complex antigens for panning of the scFv library. Merozoites were isolated from the intestinal scrapings according to Shirley [50]. The merozoite protein extracts were prepared as described above for the oocysts and sporozoites. The cell pellets resulting from preparation of soluble protein extracts of oocysts and sporozoites were used to extract the membrane proteins. Cell pellets were resuspended in extraction buffer (0.1 M Tris-HCl, pH 6.8, 5 mM EDTA, 1% Triton X-100, 1 mM PMSF) and incubated at 4°C overnight. The membrane protein fraction was separated by centrifugation for 20 min at 13,000 *g*. For antigen immobilization, Nalge/Nunc-microtiter plates were coated with 2 μg *Eimeria *extracts in PBS overnight at 4°C. After blocking with 2% BSA/PBS, 5 × 10^10 ^cfu phages were added to the wells and incubated at room temperature for 2 h. After repeated washing (16× with PBS/0.1% Tween, 4× with PBS), the bound phages were eluted with 0.1 M triethylamine, pH 12.3. The eluate was immediately neutralized with 1 M Tris-HCl, pH 7.4 and used for infection of *E. coli *XL1-Blue cells in 2xYT medium. The rescued phagemids were packaged into the phage particles by superinfection of the phagemid-containing bacteria with M13K07 helper phage in 2xYT, 2% glucose, 100 μg/ml ampicillin followed by incubation in presence of kanamycin (70 μg/ml) at 30°C overnight. Phage particles were collected from the culture medium by precipitation with 1/5 volume 20% PEG 6000/2.5 M NaCl and used for the next round of panning. In the first round of panning, 5 × 10^10^scFv phages were applied, after three rounds of panning, amplification, and re-panning, 112 clones expressing scFv were obtained that bound the oocyst extract, 64 clones which bound sporozoite protein, 39 clones that bound membrane proteins from oocysts and sporozoites and 67 clones that bound merozoite proteins.

### Bacterial expression, isolation and purification of scFvs

The selected binders were initially produced in *E. coli *bacteria carrying the pEXHAM1 phagemids encoding the corresponding scFvs. Bacterial expression was performed essentially as described [45]. Bacteria were grown at 37°C in LB medium containing 10 mM glucose and 100 μg/ml ampicillin until OD_600 _of 0.6. Expression of the recombinant protein was induced by adding IPTG to a final concentration 50 μM followed by further incubation of bacteria at 30°C for 3 h. For isolation of periplasmic extracts, the bacterial cells were harvested by centrifugation at 4°C and resuspended in 1/10 initial volume of cold spheroplast buffer (20% sucrose, 50 mM Tris-HCl, 1 mM EDTA, pH 8.0). After incubation for 20 min on ice, the spheroplasts were separated by centrifugation at 6,200 *g *at 4°C for 10 min. The supernatant containing the periplasmic extract was collected and further cleared by additional centrifugation at 30,000 *g *at 4°C for 30 min. For analyses of activity in ELISA, the periplasmic extracts were thoroughly dialyzed against PBS (50 mM KH_2_PO_4_/K_2_HPO_4_, 150 mM NaCl, pH 6.6). The antibody fragments were isolated from periplasmic extracts by immobilized metal affinity chromatography (IMAC) on Ni-NTA His-bind resin (Novagen) according to the manufacturer's instructions. Eluted fractions were analyzed for presence of antibody fragments by SDS-PAGE followed by Coomassie staining and by Western blot analysis using either anti-His-tag or anti-c-*myc *antibodies for detection. Fractions containing the target protein were pooled and prepared for ion-exchange chromatography (IEC). The protein solutions were dialyzed against an appropriate low-salt loading buffer with a pH approximately 1 pH-unit above or below the calculated isoelectric point (p*I*). The following loading buffers were used for IEC: 50 mM imidazole-HCl, pH 6.4 or pH 7.0, and 20 mM Tris·HCl, pH 8.0, for cation-exchange chromatography on Mono S 5/50 GL column (GE Healthcare Bio-Sciences AB, Uppsala, Sweden) and for anion-exchange chromatography on Mono Q 5/50 GL column (GE Healthcare), respectively. Separation was carried out using Äkta FPLC (GE Healthcare) with a linear salt gradient (0-1 M NaCl) in the loading buffer. Elution fractions of 1 ml were collected and analyzed by SDS-PAGE followed by Coomassie staining and by Western blot analysis, as mentioned above. Selected fractions were pooled and thoroughly dialyzed either against PBS for analysis of scFv activity in ELISA or against DMEM-light (1.8 mM CaCl_2 _× 2H_2_O, 5.4 mM KCl, 0.8 mM MgSO_4 _× 7H_2_O, 110 mM NaCl, 44 mM NaHCO_3_, 1 mM NaH_2_PO_4 _× 2H_2_O, pH 7.4) for the invasion inhibition assay. The protein concentrations of purified scFv preparations were determined according to Bradford [51] using the Bio-Rad Protein Assay (Bio-Rad Laboratories GmbH, Munich, Germany). If the final protein concentration was below 1 mg/ml, the preparation was further concentrated using Vivaspin-6 or -20 (5000 MW PES, Sartorius AG, Göttingen, Germany). The antigen-binding activity of scFv preparations was tested in ELISA using oocyst extracts and/or recombinant antigens. Milk powder was used as a negative control antigen. The scFv preparations were stabilized by adding 1% (w/v) bovine serum albumin (BSA), chilled in liquid nitrogen and stored at -80°C.

### *In vitro *invasion inhibition assay

The invasion inhibition assay is based on the observation that *Eimeria *sporozoites are able to invade cultured MDBK cells. The assays were performed essentially as previously described [11,52]. In brief, sporozoites from cryopreservation were thawed and washed or freshly isolated sporozoites of *E. tenella *were labeled in HBSS (Invitrogen) with 1 μM 5,6-carboxy-succinimidyl-fluoresceine-ester (CFSE, Invitrogen) for 30-60 min and washed twice with DMEM medium supplemented with 2.5% FCS (Invitrogen GmbH, Karlsruhe, Germany). The labeled sporozoites (30,000-100,000/well) were preincubated with different dilutions of purified scFv (0.5-100 μg/ml) or with the buffer control for 60 min at RT and thereafter used for infection of MDBK cells. One day prior the infection, the wells of 48-well Multidishes (Nunc, Wiesbaden, Germany) were seeded with 50,000 MDBK cells in 400 μl medium. The cells were allowed to grow for 24 hrs to 60-80% confluence in DMEM, 10% FCS. After incubation of antibody-coated sporozoites with the cells at 37°C for 4-16 hrs, the MDBK cells were washed, detached with Accutase (PAA Laboratories) and analyzed by flow cytometry using a flow cytometer CyFlow SL (Partec GmbH, Münster, Germany). The fluorescent free sporozoites as well as labeled sporozoites in infected cells were detected at 530 nm. The infected cells, non-infected cells and free sporozoites were gated using software FloMax (Partec) for subsequent counting of the infected and non-infected cells. The deduced percentages of infected cells in presence/absence of inhibitory antibody were used for calculation of the inhibition rates as follows:



### Generation of recombinant *Eimeria *antigens

As a source of genetic material, an *E. tenella *sporozoite specific cDNA library (λZAP II) [53] was used which was kindly provided by Dr. Marie Labbe (Laboratoire de Virologie et Immunologie Moléculaires INRA F 78352, Jouy-en-Josas, France). A gene encoding a 25 kDa precursor of a GPI-anchored surface antigen (*Et*SAG1; original name TA4 antigen; EMBL accession AJ586531.2) of *E. tenella *sporozoites [12] devoid of the signal and GPI-anchor sequences was amplified by PCR using a forward (sense) primer, 5'-ATG GTA GGT CTC AGG CCA TGC AGG ATT ACC CAA CAG CAG T, and a reverse (anti-sense) primer, 5'-ATG GTA GGT CTC AGC GCT GAC TGG AGA AAC TCC GCC CTT C, and cloned into *Bsa*I-digested pASK-IBA2 expression vector (IBA, Göttingen, Germany). In the generated plasmid, the *Et*SAG1 gene was placed under the transcriptional control of the tetracycline promoter/operator and was preceded by the OmpA signal sequence for secretion of the recombinant protein into the bacterial periplasm. In addition, the expression product contained a C-terminal *Strep*-Tactin affinity tag (*Strep*-tag II) for purification. Protein expression was induced in *E. coli *shaking culture by adding anhydrotetracycline to a final concentration of 200 μg/l. Affinity purification of the recombinant *Et*SAG1 antigen was performed according to the manufacturer's instruction. Alternatively, PelB leader-driven periplasmic expression of the 25 kDa *Et*SAG1 precursor with the C-terminal His_6_-tag was achieved using the bacterial expression vector pSKK3 [54] kindly provided by Affimed Therapeutics AG (Heidelberg, Germany). For cloning, the *Et*SAG1 gene was re-amplified from the previously described pASK-IBA2-based construct using the primers SAG-NcoI, 5'-ATA TTT **CCA TGG **CGG ATT ACC CAA CAG CAG, and SAG-NotI, 5'-ATG GGA TCC A**GC GGC CGC **GAC TGG AGA AAC TCC G, and cloned into *Nco*I/*Not*I-digested pSKK3. Bacterial expression in *E. coli *RV308, preparation of periplasmic extracts and antigen isolation by IMAC were performed essentially as described [55]. Fine purification of the IMAC-enriched recombinant *Et*SAG1 antigen (90-95% purity) was achieved by anion-exchange chromatography on a Mono Q 5/50 GL column (GE Healthcare) in 20 mM Tris-HCl, pH 8.0, with a 0-1 M NaCl linear gradient. Elution fractions of 1 ml were collected and analyzed by SDS-PAGE followed by Coomassie staining. The fractions containing most pure target protein were pooled and thoroughly dialyzed against PBS, pH 6.6. The recombinant *Et*SAG1 antigen was isolated with a yield of 5 mg/l bacterial culture and purity above 90%. The other seven recombinant antigens of *E. tenella *used for analyses of specificity of the library-derived antibody fragments are listed in Additional file 8. The corresponding genes were retrieved by PCR using the designed gene-specific primers and cloned into different bacterial expression vectors (Additional file 8). After sequence verification, expression in bacteria was performed according to the standard protocols [56] or to manuals of the corresponding vector manufacturer (Novagen/Calbiochem-Novabiochem GmbH, Schwalbach, Germany; IBA Göttingen GmbH, Göttingen, Germany). Depending on the vector system, the recombinant antigens were purified either by IMAC or by *Strep*-Tactin affinity chromatography.

### Analysis of binding properties and antigen specificity of selected scFvs

ELISA and Western blot analyses were performed according to the standard protocols using oocyst extracts and recombinant antigens. For IFAT and flow cytometry, sporozoite isolation was performed from freshly passaged sporulated oocysts. After excystation, the sporozoites were purified by Percoll density gradient centrifugation [57]. Cryopreservation of sporozoites was carried out as described [58]. After thawing and washing, the sample was inspected by light microscopy for the correct number of parasites. For IFAT, isolated sporozoites of *E. tenella *were air dried in Lab-Tek™ Chamber Slides™ (Nunc, 178599). For fluorescent staining of intracellular sporozoites, HepG2 cells [59] were grown in Lab-Tek™ II Chamber Slides™ (Nunc, 154534) overnight followed by infection with 30,000 sporozoites per chamber for at least 2 hrs. Sporozoites and HepG2 cells, respectively, were fixed and permeabilized by methanol treatment (90 ml methanol/10 ml 100 mM MES, 1 mM EGTA, 1 mM MgCl_2_, pH 6.9) for 5 min. After blocking for 1 h with 5% BSA-PBS, the slides were incubated with scFv preparations (5-20 μg/ml in 5% BSA-PBS, 0.05% Tween-20) for 1 h at RT. Bound antibody fragments were detected by the Alexa-555 or -488-conjugated anti-Penta-His antibody (Qiagen) (1/100 dilution in 5% BSA-PBS, 0.05% Tween-20). After each antibody incubation, the slides were extensively washed twice with PBS containing 0.05% Tween-20, and once with PBS. In addition, the nuclei of HepG2 cells were stained with DAPI (4',6-diamidin-2'-phenylindoldihydrochlorid; Invitrogen; 3 μl 0.02% stock solution per 1 ml PBS) for 15 min during the final washing procedure. After removal from the chambers, the glass slides were mounted with Aqua-Poly/Mount, water-soluble non-fluorescing mounting medium (Polysciences Europe GmbH, Eppelheim, Germany). The specimens were examined either by phase contrast light microscopy or by fluorescence microscopy with blue light (470-490 nm) using an Axioplan 2 imaging mot and the AxioCam MR (Carl Zeiss Jena GmbH, Jena, Germany). For flow cytometry, 50,000-100,000 sporozoites in each sample were incubated with the scFv-containing crude periplasmic extracts in dilutions 1/5 - 1/10 in 500 μl DMEM + 2.5% FCS for 1 h at RT. The sporozoites were washed once with 1 ml PBS, collected by centrifugation at 2,500 *g *for 5 min and the scFvs were detected by an Alexa-488-conjugated anti-Penta-His antibody (Qiagen) (1/100 dilution in DMEM + 2.5% FCS). After incubation for 1 h on ice, the relative fluorescence intensities were determined using a FACScounter (Partec).

### Transient expression of antibody fragments in tobacco leaves

The system for transient expression in tobacco was based on MagnICON™ vectors from Icon Genetics GmbH (Halle, Germany) [60,61]. For transient expression in *Nicotiana benthamiana*, a modified version of the vector pICH10990 [60] was used. A DNA sequence encoding the poly-histidine tag was assembled from oligonucleotides 10990HIS1, 5'-CTG TAT ATC T**GG ATC C**CA TCA CCA CCA TCA CCA TTA G, and 10990HIS2, 5'-AGC TAA AGC **AAG CTT **ACT AAT GGT GAT GGT GGT GAT GGG ATC CAG, and cloned into the *Bam*HI and *Hind*III sites of pICH10990, thus resulting in an intermediate plasmid pICH-M10990. The scFv genes were amplified by PCR with gene-specific forward primers (AA28-BsaI, 5'-TTT **GGT CTC **AAG GTA TGG CGG AGG TCC AGC TGC AGC AG; AB09-BsaI, 5'-TTT **GGT CTC **AAG GTA TGG CGC AGG TCC AAC TAC AGC; AB28-BsaI, 5'-TTT **GGT CTC **AAG GTA TGG CGC AGG TCC AGT TGC AGC; AD10-BsaI, 5'-TTT **GGT CTC **AAG GTA TGG CGG AAG TGA AGC TGG TGG) and universal reverse primer KM2 (5'-CTT TCC AGA CGT TAG TAA ATG), digested with *Bsa*I and *Bam*HI and cloned into the pICH-M10990 vector. The resulting constructs pICH-M1990-AA28, -AB09, -AB21, -AB28, -AD10 were used for transformation of *Agrobacterium tumefaciens *strain GV3101 followed by selection of transformed agrobacteria on YEB-Carbenicillin-Rifampicillin (YEB-Rif-Carb) dishes. For agrobacteria-infiltration of the tobacco plants, 200 ml of overnight grown scFv gene containing *Agrobacterium *cultures were sedimented by centrifugation (6,000 *g*, 3 min) and resuspended in 1 l of 10 mM MES(pH 5.5), 10 mM MgSO_4_. The resulting bacterial suspension was mixed with equal amounts of two agrobacteria strains transformed with plasmids pICH14011 (encodes DNA integrase) and pICH10570 (5'-provector for cytosolic expression), respectively, prepared in the same way. Greenhouse-grown plants of *N. benthamiana *were infiltrated by immersing the whole plant into 3 l of the resulting agrobacteria suspension followed by vacuum application (-0.8 bar) for 3 minutes. The gentle return to atmospheric pressure causes the agrobacterial suspension to spread inside the apoplastical system. The infiltrated *N. benthamiana *plants were further grown in the greenhouse. Maximum product accumulation was observed after 9-12 days, as determined by Western blot analysis of leaf discs. For large scale antibody production, the leaves of 12 plants (approx. 400 g) were collected and homogenized in an extraction buffer (50 mM KH_2_PO_4_/K_2_HPO_4_, 100 mM ascorbic acid) using a Waring Blender. The homogenate was cleared by filtration through Miracloth (Calbiochem, San Diego, CA) followed by centrifugation at 14,000 *g *and 4°C for 20 min. The antibody fragments were isolated from the clear supernatant by batch incubation with Ni-NTA His-bind resin (Novagen) and subsequent standard purification according to protocols from the manufacturer.

### Pea plant transformation and expression of scFvs in pea seeds

For transformation of pea plants, an expression system based on the binary vector pPZP200 [62] with a seed specific promoter, regulation signals from *Vicia faba *[63-65] and a 35S terminator of transcription from cauliflower mosaic virus (CaMV) [66] was used. The key features of the vector are outlined in Additional file 9. The scFv genes were cloned into the seed specific expression cassette of the pPZP200-USP+ plasmid using *Nco*I and *Bam*HI restriction sites (Additional file 9) followed by electroporation into strain EHA105 of *A. tumefaciens*. For transformation, the fodder pea variety "Eiffel" was chosen. Pea transformation was carried out as described [67] with modifications [36]. In brief, immature pods were sterilized in 70% (v/v) ethanol (1 min) followed by 1% (w/v) sodium hypochlorite (20 min) and three washes with sterile distilled water. Seeds were removed from the pods and the outer coats (*testae*) of the seeds were excised. Coatless seeds were pre-cultivated for 1 day in liquid B_5_h medium [68]. For preparation of explants, the root end of each segment was cut off and the epicotyl and the apical meristem regions were sliced transversely into 3-5 segments. Segments were then fully immersed in the agrobacterial suspension (10^8 ^cells/ml) for 30 to 40 min. The suspension comprised two strains of transformed *Agrobacterium*, one carried the plasmid pPZP200-USP-scFv (see above) and the second one contained a binary vector pPZP-bar with the marker gene *bar *encoding phosphinotricin acetyltransferase (PAT), which confers resistance to gluphosinate ammonium (herbicide Basta). Wet segments were plated on B_5_h medium and cultured at 21°C for 3-4 days with a 16 h photoperiod. After co-cultivation, explants were washed three to four times with sterile water. The callus induction medium and the shoot development were as described by Schroeder *et al*. [67]. When the developing shoots reached over 20 mm in length, they were grown on 10 mg/L phosphinotricin for some days. Resistant shoots were grafted onto the root stock of "Eiffel" seedlings *in vitro*. After 6-10 days, the plants were adapted to soil and grown up in a climatic chamber. When primary transformants (F_0_) developed to a stage of 4-6 normal nodes, leaflets were painted with a 0.5% (v/v) watery solution of Basta (Sanofi-Aventis) on the upper side to determine the *bar *transgenic plants. Integration of the scFv transgene was checked by PCR in leaflet extracts using the primers scf-uni-up, 5'-AAA TTC ACC TTC TTC AAG C, and USP-do, 5'-AGG TGC ATG AAC GTC ACG TGG. The F_0 _plants were allowed to self-pollinate and 10-20 seeds were analyzed for presence of the scFv gene by PCR and for expression of the scFv protein by SDS-PAGE and Western blot analysis. Using segregation analysis for both scFv and *bar *genes, non-coupled single insert lines for the scFv gene were determined. From all progeny of a single insert line, with the out-segregated *bar *gene, the homozygous state of the scFv gene was tested in the next (F_2_) generation. Thus, co-transformation of both the bar and the scFv gene allowed selection of transgenic plants with no resistance (*bar*) gene. For estimation of the expression levels, the scFv antibody fragments were isolated from the pea seeds and purified. For isolation of the recombinant protein, the pea seeds were ground using a ball mill and the resulting pea flour was suspended in ice-cold extraction buffer containing 50 mM Tris-HCl, 300 mM NaCl, 0.2% Tween-20, pH 7.4, at a ratio of 1/20 (w/v). Extraction was performed at 4°C in three steps. After each step, the samples were centrifuged at 30,000 *g *and 4°C for 30 min. The supernatants were pooled and subjected to IMAC on Ni-NTA His-bond resin (Novagen) using the extraction buffer as loading and elution buffer, in the latter case supplemented with 500 mM imidazole. The target protein was further purified using IEC and analyzed, as mentioned earlier for bacterial and tobacco scFv expression. Purified scFv was used as a standard for the quantification of antibody expression in pea seeds by ELISA using *Eimeria *oocyst extract as an antigen. Titration of scFv standard generated a calibration curve from which the amounts of active antibody in diluted pea seed extracts were deduced. Extracts from the pea seeds expressing scFv AB28 were prepared by grinding the seeds in a ball mill followed by soluble protein extraction from a defined amount of the powder in PBS containing 1 mM PMSF. As a negative control, extracts from wild-type pea seeds prepared in the same way were used. Clear supernatants were applied to an ELISA plate in serial dilutions. Detection was performed using anti-penta-His antibody (Qiagen) followed by HRP-conjugated anti-mouse IgG (Sigma) and TMB as a substrate. Data were analyzed using the software Prism (GraphPad, San Diego, CA). The purity of the scFv standard, as determined by scanning a Coomassie-stained SDS-PAA gel, was considered in the calculations.

### Analysis of the proteolytic and pH stability *in vitro*

For analyses of the pH stability, commercially available standard buffers of pH 2-11 (Roth, Karlsruhe, Germany) were used. Analyses were performed by ELISA using plates coated with the diluted extract from *E. tenella *oocysts (0.4 μg/well). In the binding assays, the antibody fragments were diluted in buffers with different pH, applied to the antigen-coated ELISA plate and incubated for 1 h at RT. For dissociation analyses, the antibody samples were applied in PBS/2% BSA, pH 6.6, and after incubation (1 h at RT) and washing the buffer solution of a defined pH was added followed by 1 h incubation. In all cases, the bound antibody fragments were detected using anti-penta-His antibody (Qiagen). For measuring the proteolytic stability, an "*In vitro *true digestibility" protocol from Ankom Technology (Macedon, NY) was used. For the experiments, chyme was taken from the small intestine of chickens, cleared by centrifugation and the resultant intestinal fluid was stored in aliquots at -80°C. Before start of the experiment, buffer solutions A and B were prepared according to the following recipes. The buffer solution A comprised 10 g/l KH_2_PO_4_, 0.5 g/l MgSO_4 _× 7H_2_O, 0.5 g/l NaCl, 0.1 g/l CaCl_2 _× 2H_2_O and 0.5 g/l urea (reagent grade). Respectively, the buffer solution B contained 15.0 g/l Na_2_CO_3 _and 1.0 g/l Na_2_S × 9H_2_O. The buffer solutions A and B were pre-warmed to 39°C and mixed in a ratio of 5/1 to obtain pH 6.8. No further pH adjustment was necessary. The resulting buffer AB was used to prepare a series of 1/2 dilutions of the chicken intestinal fluid. The antibody fragments were incubated with the dilutions of intestinal fluid at 37°C for 30 min on a rocking platform. The residual antigen-binding activity was measured by ELISA using plates coated with oocyst extract of *E. tenella*. In addition, protein degradation was monitored by SDS-PAGE followed by Coomassie staining and by Western blot analysis with the anti-His-tag detection. For quantification of the results, the *ED*_50 _values (effective dilution leading to 50% loss of activity) were deduced from the sigmoid ELISA curves [log(intestinal fluid dilution) vs. response (% maximal binding)] by fitting them using a standard slope model of software Prism (GraphPad).

### Force-feeding animal trials

Studies were performed using 10-day old non-sexed commercial Lohmann broiler chickens (type Ross) with an approx. weight of 100 g randomly divided into groups of 3-30 individuals (the cohort size varied in different studies). The chickens were orally infected with a single dose of *E. tenella *freshly prepared oocysts (20-500 oocysts per bird depending on the study). Force-feeding started one day before infection and continued for 8 consecutive days. Birds in the treatment groups received either 1 mg scFv isolated from tobacco leaves or transgenic pea seed flour in 1 ml PBS a day via gavage. The chickens were force-fed three times daily. The maximum dose of pea seeds which could be safely administered to a 10-day old chicken was empirically determined as one pea seed a day or 1/3 pea seed per administration. The pea flour was prepared from the antibody containing seeds using a ball mill and was suspended in phosphate buffer just before force-feeding. Based on the activity measurements in ELISA, the AB28 antibody content in pea seed was calculated as 1.76 g scFv/kg seeds. Assuming the average weight of a pea seed as 0.3 g, the pea flour-fed birds received the maximal possible dose of 0.5 mg antibody per day (half the amount of administered purified scFv). As positive controls of infection, two animal groups were treated with the same quantities of material comprising the irrelevant BA11 antibody, either in the form of pea flour or as a soluble antibody isolated from the tobacco leaves. The negative control of infection group received heat inactivated oocysts. For comparison of anti-coccidial effect, one group received 1 ml of a 2.5% solution of the anti-coccidial drug Baycox^® ^(Bayer HealthCare) in water. Baycox^® ^is currently used to control *Eimeria *in commercial poultry farming. The treatment started one day before the infection and continued for 14 consecutive days. For all animals, fecal samples were collected before infection (to show whether the animals were already infected before the start of the experiment) and daily after infection. The oocyst counts were determined in all individual fecal samples and each sample was counted six times by two different persons. In addition, the chicken weights were controlled and recorded. At the end of each study, all birds were sacrificed and weighted according to the protocol. One individual of each group was subjected to necropsy. As measures of infection outcome, the sum of shed oocyst numbers at days of maximum oocyst release, 7 and 8 p.i., were used. To illustrate the specific effect of the anti-*Eimeria *treatment, the percentage of infection inhibition was calculated according to the following formula:



### Ad libitum feeding trials

In a preliminary feeding experiment, twenty 9-day-old chickens were fed with normal feed (n = 10) and with the feed premixed with 10% BA11-transgenic pea shred (n = 10) for 8 days. The feed was weighed and reweighed every day, the body weight was recorded and the feces were collected on feeding days 3 and 7. The antibody content in the fodder and in feces was controlled by the BA11-specific ELISA. In a high infection dose model, the broiler chickens were orally infected with individual doses of 2,500 freshly prepared oocysts. Three treatment cohorts were formed; each comprising 5-30 individually housed ten-day old chickens with average weight of 127.5 g. For feeding, the usual chicken fodder was mixed with 10% pea shred derived either from AB28-expressing seeds (treatment group, n = 30) or from the wild-type pea (positive control of infection, n = 25). For shredding, the pea seeds were coarsely ground with a centrifugal mill ZM 1000 (Retsch, Haan, Germany). The negative control group (control of spontaneous infection) comprised 5 birds which were not infected and received conventional fodder. Feeding started one day before the infection and continued for eight consecutive days. Adapted from the preliminary feeding experiment, the mean consumption of the scFv antibody was calculated as 35.63 mg antibody/kg body weight × day (Additional file 7). On the seventh day p.i., the chickens were sacrificed and the caecal lesion scores and the oocyst counts in caeca and feces were determined. The low infection dose model was designed to mimic the real situation in the barns for rearing commercial broilers where the chickens are spontaneously infected with the relatively low dosages of *Eimeria *oocysts. In this case, the feeding experiment was repeated under the same conditions, however, with the ten-fold lower infection doses, i.e. 250 oocysts per bird.

### Statistical analysis

Data analyses were performed using the software Prism (GraphPad). All data were expressed as means ± SEM values. Comparisons of the mean values were performed between the control and treatment groups using a one-tail unpaired *t*-test.

## Competing interests

The authors declare that they have no competing interests.

## Authors' contributions

JZ and KZ carried out library generation and screening. IS led generation of the transgenic pea plants. DJ carried out antibody characterization *in vitro *and supervised animal experiments. MG led gene cloning and plasmid constructions. SH led protein purification and stability analyses. JW and GG led the animal experiments. JM performed characterization of transgenic pea plants. DF initiated the work. SMK led the work and wrote the manuscript. All authors read and approved the final manuscript.

## Supplementary Material

Additional file 1 **Characterization of AB28-transgenic pea lines**.: The data provided represent PCR and Western blot analyses of F_0 _and F_1 _transgenic pea plants. Click here for file

Additional file 2 **Analyses of independent AB28 F_0 _pea lines**. The data provided represent a summary of basta selection, PCR and Western blot analyses of independent F_0 _transgenic pea lines. Click here for file

Additional file 3 **Analyses of F_1 _seeds derived from AB28 F_0 _pea line 9**. The data provided represent a summary of PCR and Western blot analyses of F_1 _seeds derived from AB28 F_0 _pea line 9. Click here for file

Additional file 4 **Results of analyses of the progeny of F_1 _lines 9/9, 9/10, 9/11 and 9/13**. The data provided represent a summary of Western blot analyses of F_2 _seeds derived from different AB28 F_1 _lines. Click here for file

Additional file 5 **Characterization of purified scFv AB28 produced in pea seeds**. The data provided represent analysis of the molecular forms of pea-derived scFv and head-to-head comparison of the antigen-binding properties of the scFv AB28 preparations isolated either from the tobacco leaves or from the transgenic pea seeds. Click here for file

Additional file 6 **Preliminary *ad libitum *feeding experiment**. The data provided represent analyses of the body weight and the feed consumption by chickens fed *ad libitum *with the fodder containing either transgenic or wt pea together with the assessment of shunning transgenic pea shred. Click here for file

Additional file 7 **Feed consumption and antibody uptake in chickens**. The data provided represent measured average feed consumption, body weight gains and calculated antibody uptake by chickens in a preliminary feeding experiment. Click here for file

Additional file 8 **List of expressed recombinant antigens of *Eimeria tenella*, their gene accession numbers and used PCR primers**. The file provides information about generation of recombinant antigens used in the present study. Click here for file

Additional file 9 **Characteristics of the binary vector used for pea transformation**. The file provides information about the genetic elements included into the binary vector used for pea transformation. Click here for file
