# Supplementary material for: Antibody expressing pea seeds as fodder for prevention of gastrointestinal parasitic infections in chickens
Source: BMC Biotechnol. 2009 Sep 11;9:79. doi: 10.1186/1472-6750-9-79 (PMC2755478; doi:10.1186/1472-6750-9-79)
Supplement: Additional file 1 — Characterization of AB28-transgenic pea lines.: The data provided represent PCR and Western blot analyses of F0 and F1 transgenic pea plants. [file 1472-6750-9-79-S1.pdf]

Additional file 1. Characterization of AB28-transgenic pea lines.

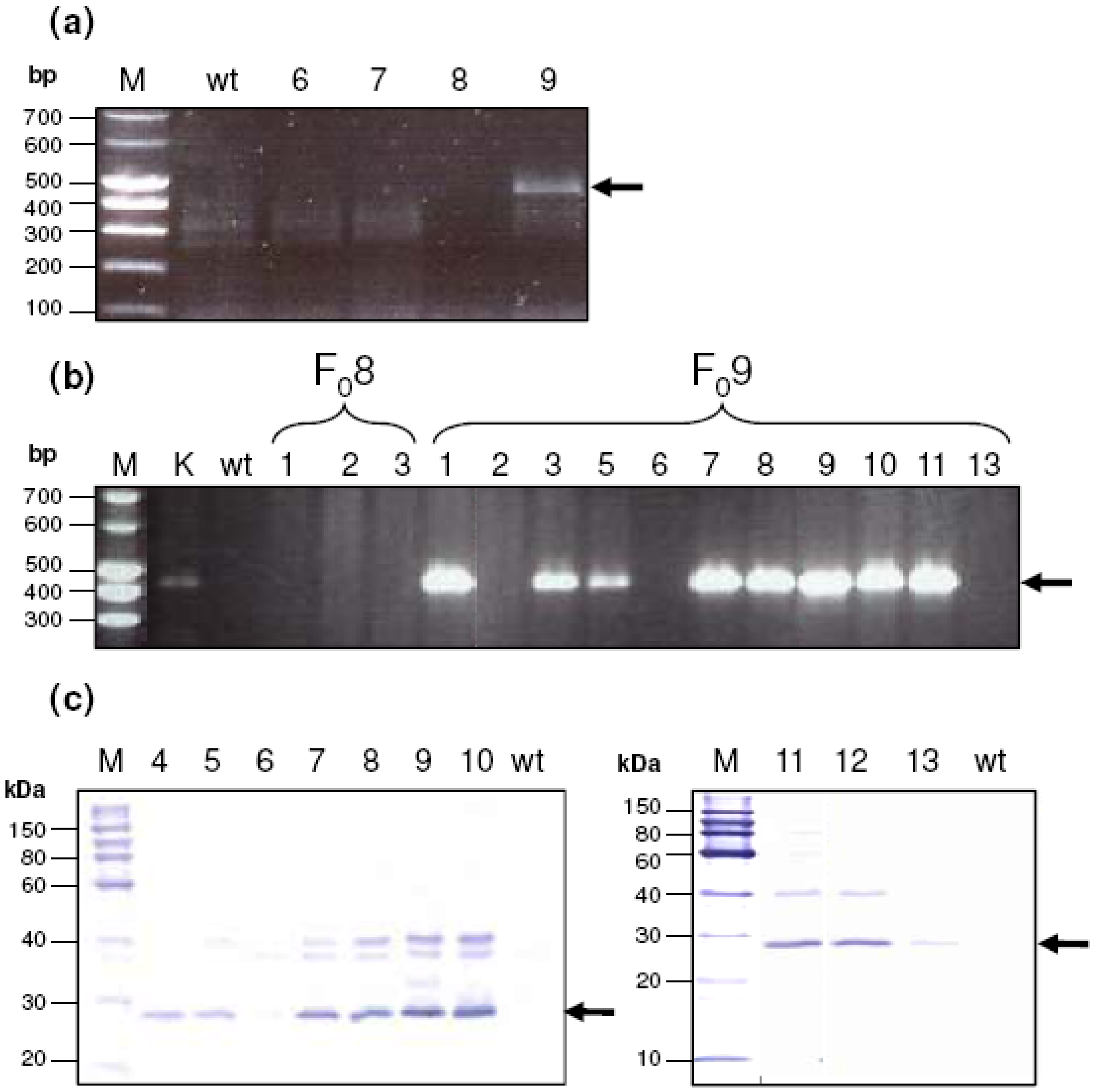

(a) PCR analysis of the leaf material from F_0 plants 6, 7, 8 and 9. Lanes: M, SmartLadder SF DNA marker (Eurogentec, Liege, Belgium); wt, sample from non-transgenic pea plant. **(b)** PCR analysis of the F_1 transgenic plants derived from F_0 plants 8 and 9. Lanes: K, positive control; M, SmartLadder SF DNA marker (Eurogentec); wt, sample from the non-transgenic pea plants. The positions of the specific DNA bands of 450 bp are indicated. **(c)** Western blot analysis of F_1 seeds derived from the F_0 plant 9 for scFv expression. Lanes: M, molecular weight markers (10-150 kDa PLUS, Serva); wt, samples from non-transgenic seeds; 4-13, samples of the accordingly numbered seeds. Positions of specific protein bands of approx. 25 kDa are indicated with arrows.
