# Supplementary material for: Antibody expressing pea seeds as fodder for prevention of gastrointestinal parasitic infections in chickens
Source: BMC Biotechnol. 2009 Sep 11;9:79. doi: 10.1186/1472-6750-9-79 (PMC2755478; doi:10.1186/1472-6750-9-79)
Supplement: Additional file 2 — Analyses of independent AB28 F0 pea lines. The data provided represent a summary of basta selection, PCR and Western blot analyses of independent F0 transgenic pea lines. [file 1472-6750-9-79-S2.pdf]

Additional file 2. Analyses of independent AB28 F_0 pea lines.

**No. independent F_0 line**	**Basta-selection**	**PCR**	**Western blot analysis (His-tag) of F_1 seeds**
1	(-)	(-)	(-)
2	(+)	(-)	(-)
3	(+)	(-)	(-)
4	(+)	(-)	(-)
5	(+)	(-)	(-)
6	(+)	(-)	(-)
7	(+)	(-)	(-)
8	(-)	(-)	(-)
9*	(+)	(+)	(+)
10	(+)	(-)	(-)

*Line 9 has been selected for further work. (-), negative; (+), positive.
