# Supplementary material for: Antibody expressing pea seeds as fodder for prevention of gastrointestinal parasitic infections in chickens
Source: BMC Biotechnol. 2009 Sep 11;9:79. doi: 10.1186/1472-6750-9-79 (PMC2755478; doi:10.1186/1472-6750-9-79)
Supplement: Additional file 3 — Analyses of F1 seeds derived from AB28 F0 pea line 9. The data provided represent a summary of PCR and Western blot analyses of F1 seeds derived from AB28 F0 pea line 9. [file 1472-6750-9-79-S3.pdf]

Additional file 3. Analyses of F_1 seeds derived from AB28 F_0 pea line 9.

**F_1 seed No.**	**PCR**	**Western blot analysis (His-tag) of F_1 seeds**
1	(+)	(+)
2	(-)	(-)
3	(+)	(+)
4	(+)	n.d.
5	(+)	(+)
6	(-)	(-)
7	(+)	(+)
8	(+)	(+)
9	(+)	(+)
10	(+)	(+)
11	(+)	(+)
12	(+)	n.d.
13	(-)	(-)

(-), negative; (+), positive; n.d., not determined.
