# Supplementary material for: Antibody expressing pea seeds as fodder for prevention of gastrointestinal parasitic infections in chickens
Source: BMC Biotechnol. 2009 Sep 11;9:79. doi: 10.1186/1472-6750-9-79 (PMC2755478; doi:10.1186/1472-6750-9-79)
Supplement: Additional file 4 — Results of analyses of the progeny of F1 lines 9/9, 9/10, 9/11 and 9/13. The data provided represent a summary of Western blot analyses of F2 seeds derived from different AB28 F1 lines. [file 1472-6750-9-79-S4.pdf]

Additional file 4. Results of analyses of the progeny of F_1 lines 9/9, 9/10, 9/11 and 9/13.

No. F_1 pea line	F_2 seeds	Western blot analysis (His-tag) of F_2 seeds	Comment
**9/9***	1	(+)	**homozygous**
	2	(+)	
	3	(+)	
	4	(+)	
	5	(+)	
	6	(+)	
**9/10**	1	(+)	**heterozygous**
	2	(-)	
	3	(+)	
	4	(+)	
	5	(+)	
	6	(-)	
**9/11**	1	(+)	**heterozygous**
	2	(+)	
	3	(-)	
	4	(+)	
	5	(-)	
	6	(+)	
**9/13**	1	(-)	**negative**
	2	(-)	
	3	(-)	
	4	(-)	

*For line 9/9, 16 tested seeds were positive. (-), negative; (+), positive.
