# Supplementary material for: Antibody expressing pea seeds as fodder for prevention of gastrointestinal parasitic infections in chickens
Source: BMC Biotechnol. 2009 Sep 11;9:79. doi: 10.1186/1472-6750-9-79 (PMC2755478; doi:10.1186/1472-6750-9-79)
Supplement: Additional file 5 — Characterization of purified scFv AB28 produced in pea seeds. The data provided represent analysis of the molecular forms of pea-derived scFv and head-to-head comparison of the antigen-binding properties of the scFv AB28 preparations isolated either from the tobacco leaves or from the transgenic pea seeds. [file 1472-6750-9-79-S5.pdf]

Additional file 5. Characterization of purified scFv AB28 produced in pea seeds.

(a)

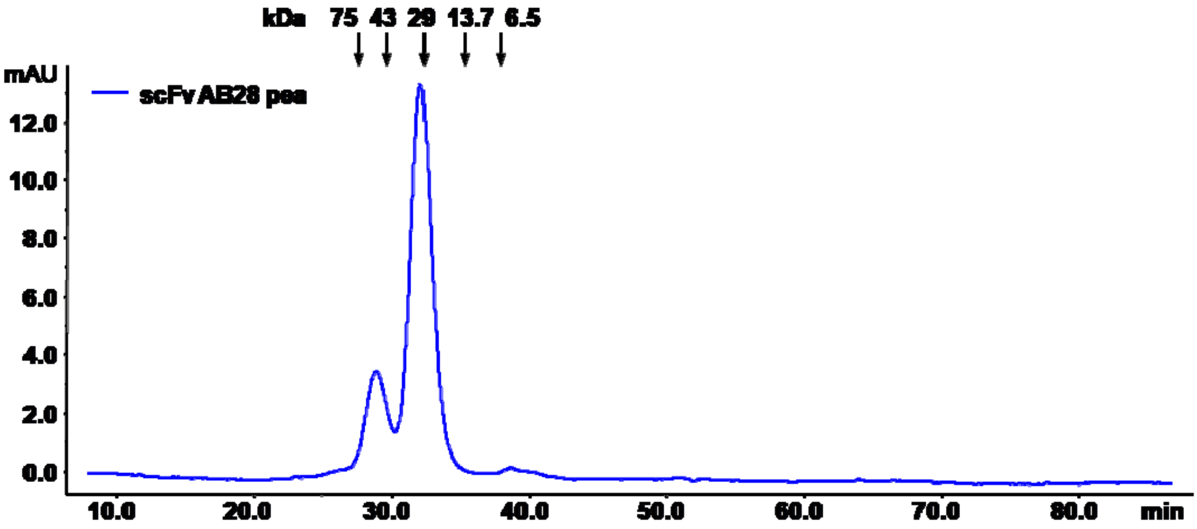

(b)

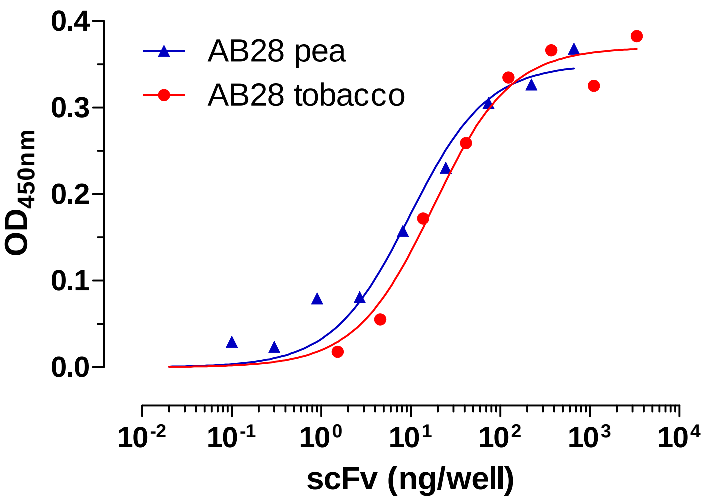

(a) Analysis of the molecular forms by size-exclusion FPLC on a calibrated Superdex 200 column. The positions of the molecular weight markers are indicated. **(b)** Head-to-head comparison of the antigen-binding activities of the scFv AB28 preparations isolated either from the tobacco leaves or from the transgenic pea seeds, as measured by ELISA.
