# Supplementary material for: Antibody expressing pea seeds as fodder for prevention of gastrointestinal parasitic infections in chickens
Source: BMC Biotechnol. 2009 Sep 11;9:79. doi: 10.1186/1472-6750-9-79 (PMC2755478; doi:10.1186/1472-6750-9-79)
Supplement: Additional file 6 — Preliminary ad libitum feeding experiment. The data provided represent analyses of the body weight and the feed consumption by chickens fed ad libitum with the fodder containing either transgenic or wt pea together with the assessment of shunning transgenic pea shred. [file 1472-6750-9-79-S6.pdf]

Additional file 6. Preliminary *ad libitum* feeding experiment.

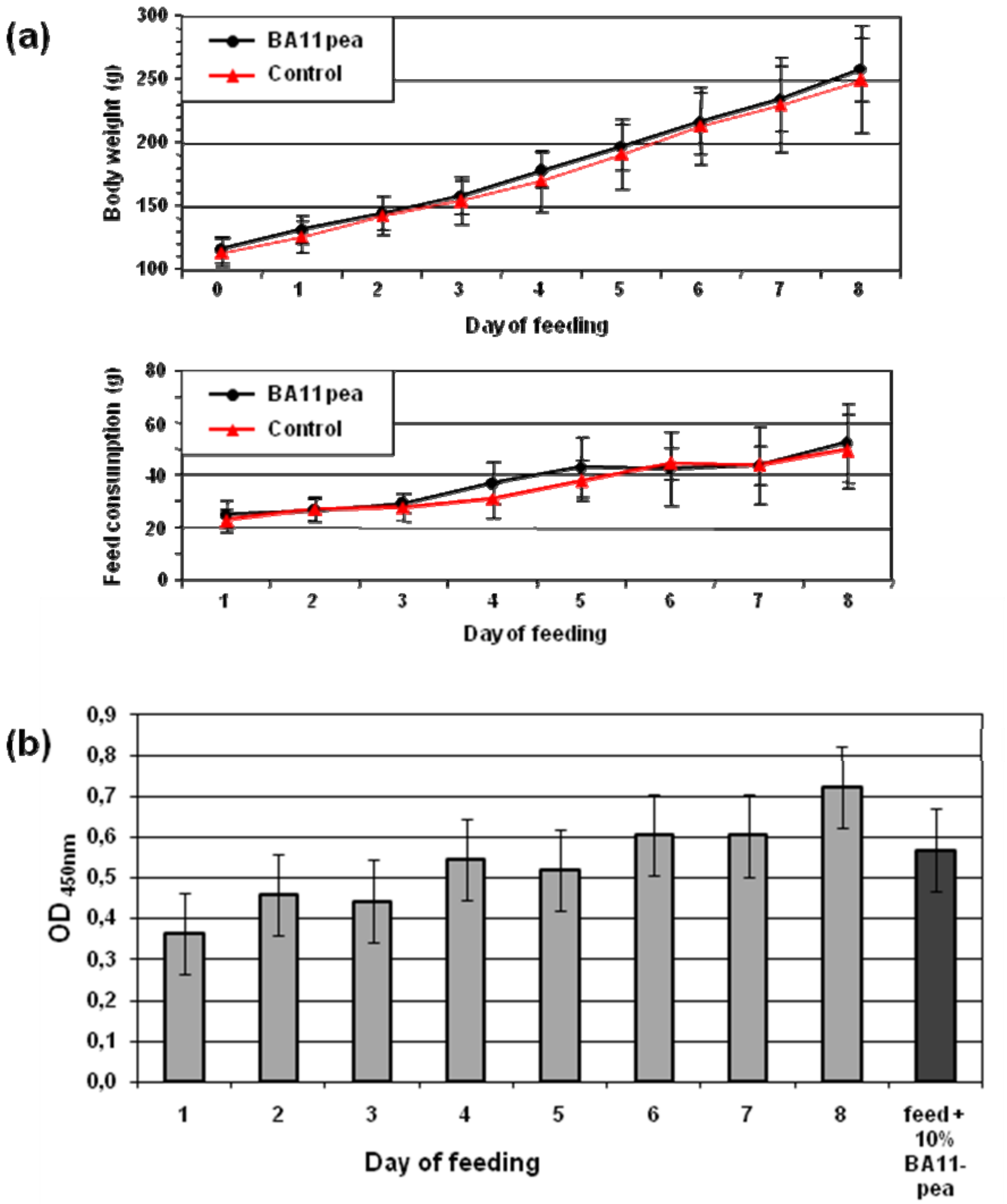

Two chicken cohorts were fed *ad libitum* with the fodder containing either BA11-transgenic pea or wt pea. **(a)** Analyses of the body weight and the feed consumption per animal in each group. Means and SDs are plotted against days of feeding. **(b)** Assessment of shunning transgenic pea shred in preliminary feeding experiment. After feeding, the residual feed was collected, weighted and used for extraction of soluble protein. Diluted extracts were used in ELISA for analysis of antigen-binding activity as a measure of antibody content. For comparison, the original feed with 10% BA11-pea shred was used.
