# Supplementary material for: Antibody expressing pea seeds as fodder for prevention of gastrointestinal parasitic infections in chickens
Source: BMC Biotechnol. 2009 Sep 11;9:79. doi: 10.1186/1472-6750-9-79 (PMC2755478; doi:10.1186/1472-6750-9-79)
Supplement: Additional file 7 — Feed consumption and antibody uptake in chickens. The data provided represent measured average feed consumption, body weight gains and calculated antibody uptake by chickens in a preliminary feeding experiment. [file 1472-6750-9-79-S7.pdf]

Additional file 7. Feed consumption and antibody uptake in chickens.

**Age**	**Day of feeding**	**Average feed consumption (g)**	**Pea content (g)**	**Average antibody uptake (mg/bird*d)**	**Average body weight (g)**	**Average antibody uptake (mg/kg bw*d)**
Day 9	Day 1	23.70	2.37	4.17	114.62	36.39
Day 10	Day 2	24.70	2.47	4.35	127.94	33.97
Day 11	Day 3	26.43	2.64	4.65	143.17	32.49
Day 12	Day 4	32.63	3.26	5.74	155.80	36.86
Day 13	Day 5	37.68	3.77	6.63	174.08	38.09
Day 14	Day 6	40.58	4.06	7.14	194.12	36.79
Day 15	Day 7	41.70	4.17	7.34	214.48	34.22
Day 16	Day 8	47.81	4.78	8.41	232.44	36.20

The calculations are based on the determined AB28-content of 1.76 mg extractable functional scFv AB28 per 1 g dry seed weight.
