# Supplementary material for: Antibody expressing pea seeds as fodder for prevention of gastrointestinal parasitic infections in chickens
Source: BMC Biotechnol. 2009 Sep 11;9:79. doi: 10.1186/1472-6750-9-79 (PMC2755478; doi:10.1186/1472-6750-9-79)
Supplement: Additional file 8 — List of expressed recombinant antigens of Eimeria tenella, their gene accession numbers and used PCR primers. The file provides information about generation of recombinant antigens used in the present study. [file 1472-6750-9-79-S8.pdf]

Additional file 8. List of expressed recombinant antigens of *Eimeria tenella*, their gene accession numbers and used PCR primers.

***E. tenella* antigen**	**GenBank accession**	**Expression vector**	**Restriction sites for cloning**	**PCR primers**
*Et*Mic1 (microneme protein) [13]	AF032905<br>M73495A	pET16b (Novagen)	*Nde*I-*Bam*HI	Mic1-NdeI<br>Mic1-BamHI
*Et*Mic2 (microneme protein) [14]	Z71755	PET21(+) (Novagen)	*Nde*I-*Eag*I	NdeI-Mic2<br>EagI-Mic2
*Et*Mic3 (microneme protein) [11]	AY512381	pET16b (Novagen)	*Nde*I-*Bam*HI	Mic3-NdeI<br>Mic3-BamHI
*Et*Mic5 fragment (microneme protein) [15]	AJ245536	pET22b(+) (Novagen)	*Nco*I-*Xho*I	Mic5-Nco2<br>Mic5-Xho2
Eimepsin (aspartyl proteinase) [16]	AJ293829	pET22b(+) (Novagen)	*Eco*RI-*Xho*I	Eimep-EcoRI<br>Eimep-Xho2
MZP 5-7 (merozoite antigen) [17]	L08257	pET22b(+) (Novagen)	*Nco*I-*Xho*I	MZP-XhoI<br>MZP-BamHI
19 kDa Ag 3-1E (potential immunostimulator) [18]	AY745810	pASK-IBA2 (IBA)	*Bsa*I	3-1E Forward<br>3-1E Reverse

PCR primers:

Mic1-NdeI	5’- AAGGTCGT**CATATG**GCGCCCCTTCCTCG
Mic1-BamHI	5’- TTAT**GGATCC**TTAGGATGCCCACATCTCTG
NdeIMic2	5’- CATTC**CATATG**GCTCGAGCG
EagIMic2	5’- GTCC**CGGCCG**ATGACTGTTGAGTGTCAC
Mic3-NdeI	5’- TCATTATT**CATATG**CGACTGAGCAGAAGTTTC
Mic3-BamHI	5’- CATTT**GGATCC**TCACAATGTGGCCCTCTCCCC
Mic5-Nco2	5’- ATTGG**CCATGG**TGGAGACACAGTGG
Mic5-Eco2	5’- GTGCACGAGGTACCCGAATTCCCTGCG
Eimep-EcoRI	5’- TCC**GAATTC**ATGCGTTCCCTTCTGGTCGTGG
Eimep-Xho2	5’- TAT**CTCGAG**AAAGGCCAGTGCGATTGC
MZP-XhoI	5’- CGCT**CTCGAG**CTCGTCGTCCTCGTCGTCTCC
MZP-BamHI	5’- AC**GGATCC**GATGGCTAAGTCTATG
3-1E Forward	5’- ATGGTAGGTCTCAGGCCGGAGAAGCAGACACCCAGGCC
3-1E Reverse	5’- ATGGTAGGTCTCAGCGCTGAAGCCGGACTGGTGAAGGTAC
