# Supplementary material for: Antibody expressing pea seeds as fodder for prevention of gastrointestinal parasitic infections in chickens
Source: BMC Biotechnol. 2009 Sep 11;9:79. doi: 10.1186/1472-6750-9-79 (PMC2755478; doi:10.1186/1472-6750-9-79)
Supplement: Additional file 9 — Characteristics of the binary vector used for pea transformation. The file provides information about the genetic elements included into the binary vector used for pea transformation. [file 1472-6750-9-79-S9.pdf]

Additional file 9. Characteristics of the binary vector used for pea transformation.

Genetic element	Size (bp)	Description
pPZP200	6741	High copy binary vector used for cloning of DNA sequences [62]
USP+	1191	Elongated version of the Unknown Seed Protein (USP) promoter from *Vicia faba* [65]
pPZP-USP+scFv	variable	Binary vector for plant transformation (target gene encodes scFv)
UTR-LeB4	17	5'-untranslated region from legumin gene of *Vicia faba* [64]
LeB4	75	Signal sequence for legumin from *Vicia faba* which targets to the endoplasmic reticulum (ER) [64]
HIS	18	Synthetic hexa histidine tag for purification purposes
KDEL	12	Synthetic ER-retention signal
35S terminator	225	Terminator (poly A signal) from CaMV *35S* transcript [66]
